# Comparative Analysis of Commercial Organic Fertilizers Revealing Elevated Nutrient and Contaminant Loads in Pig Manure-Based Fertilizer

**DOI:** 10.3390/microorganisms14102364

**Published:** 2026-10-15

**Authors:** Na Li, Jing Ye, Chaoyang Wang, Changxiong Zhu, Hongna Li

**Affiliations:** 1 Institute of Environment and Sustainable Development in Agriculture, Chinese Academy of Agricultural Sciences, Beijing 100081, China; yejing@caas.cn (J.Y.); 15116946231@163.com (C.W.); 2 College of Environmental Sciences and Engineering, Hebei University of Science and Technology, Shijiazhuang 050018, China; zhuchangxiong@126.com

**Keywords:** manure-based fertilizers, antibiotics, ARGs, ARB, heavy metals

## Abstract

Organic fertilizers are rich in nutrients but also frequently contain multiple contaminants that pose a potential threat to the agricultural environment. However, comprehensive investigations into commercial organic fertilizers remain scarce. In this study, 54 commercial organic fertilizer samples—including animal manure-based fertilizers, BOF (bio-organic fertilizer), and other types of fertilizers—were systematically investigated. For OM (organic matter), 48.15% of samples fell below the 30% *w*/*w* threshold specified by the industry standard NY/T 525-2021. The exceedance rates of Cu and Zn across all fertilizers were 14.81% and 12.96%, respectively, relative to the German Biowaste Ordinance (BioAbfV) limits. Differences in properties among geographic origins were less pronounced than those among fertilizer types. Pig manure-based fertilizers had the highest nutrient contents, especially phosphorus, but also the highest levels of contaminants, including antibiotics, heavy metals, antibiotic-resistant bacteria (ARB), and antibiotic resistance genes (ARGs). BOF exhibited the best overall quality, with a total nitrogen content 2.0–4.4 times that of the manure-based fertilizers, together with higher organic matter and lower contaminant levels. This study establishes a comprehensive baseline for commercial organic fertilizers in China, providing critical empirical evidence for future industry governance and fertilizer management.

## 1. Introduction

Organic fertilizers, derived from animal manure, crop residues, and other organic wastes crop residues, are the main feedstocks of organic fertilizers. These fertilizers are applied to improve soil structure, replenish soil organic matter, and replace part of synthetic fertilizer inputs [1,2]. In China, the largest livestock and poultry producer in the world [1,3], the “organic fertilizer substitution for chemical fertilizer” program and related policies have promoted the conversion of manure into commercial organic fertilizers [1,2]. Such products are now widely used on croplands across the country. In 2025, organic fertilizers were applied to >6.5 × 10^8^ mu·times (≈4.33 × 10^7^ ha·times) of cropland in China [4].

Chinese livestock production is dominated by intensive pig, poultry, cattle, and sheep systems [1,3]. It is estimated that China produces about 3.8 billion tonnes of livestock and poultry manure each year, and the share of manure from large-scale farms reached 73.2% in 2023 [1]. In these large-scale farms, antibiotics and copper/zinc (Cu/Zn) supplements are added to feed for disease control and growth promotion, a large fraction of which is excreted and accumulate in the manure [5]. Moreover, organic fertilizer application can increase the abundance of antibiotic-resistant bacteria (ARB) and antibiotic resistance genes (ARGs), which have been recognized as emerging environmental contaminants [6]. These contaminants ultimately threaten human health, particularly through the consumption of crops grown in contaminated soils. This is because ingestion of plant products grown in contaminated soils is a plausible exposure route of ARGs or ARB. Once dietary ARGs are ingested, they can be disseminated among gut bacteria by conjugation, transduction and transformation [7], and ARGs carried on mobile genetic elements that also replicate in human pathogens are regarded as an immediate threat to the successful treatment of clinical infections [7,8]. Bacteria entering the gut via food can share their ARGs with resident commensal and pathogenic species by the same processes [9].

Manure is handled through three main pathways before it is applied or sold. Aerobic composting is the main route to commercial organic fertilizers; anaerobic digestion produces biogas and digestate, and direct land application, though declining, carries the highest risk [1,10]. At thermophilic temperatures (≈55–65 °C), composting reduces pathogens and a large proportion of ARGs but does not remove heavy metals; residual antibiotics together with Cu/Zn can co-select for ARGs, so ARGs can persist in compost despite thermophilic treatment [10]. The heavy metals and antibiotics already in the feedstock frequently survive this processing and pass into China’s commercial organic fertilizers [11]. These residues also survive the other routes, including anaerobic digestate and raw manure. Therefore, the final product therefore inherits a large share of its contaminant burden from the upstream farming and treatment stages. Thus, it is of vital importance to assess and ensure the safety of organic fertilizers.

Although previous studies have investigated individual classes of contaminants in organic fertilizers—such as antibiotics [12], heavy metals [13], or ARGs [14]—few have simultaneously evaluated nutrients, chemical contaminants, and microbiological hazards in commercially available products. This gap limits a comprehensive understanding of the trade-offs between fertilizer quality and environmental risk across different fertilizer types. We hypothesize that (1) the nutrient content and contaminant burden of commercial organic fertilizers vary significantly with both fertilizer type and geographic origin—pig manure-based fertilizers are expected to carry the highest pollutant loads owing to intensive farming practices, whereas regional differences in husbandry intensity, feed-additive and veterinary-antibiotic use, and composting standards are expected to further shape these profiles. (2) Bio-organic fertilizers (BOFs), produced through more controlled fermentation processes, achieve higher nutrient contents at lower contaminant burdens than conventional commercial organic fertilizers. To test these hypotheses, this study comprehensively determined nutrients, physicochemical characteristics, antibiotics, heavy metals, antibiotic-resistant bacteria (ARB), and ARGs in 54 commercial organic fertilizers across China. The findings will help stakeholders to understand the current quality of commercial organic fertilizers and provide a reference with which policy makers might establish appropriate regulations.

## 2. Materials and Methods

### 2.1. Sample Collection, Storage, and Processing

Commercial organic fertilizers produced from November 2023 to May 2024 were purchased in China either online or from agricultural supply stores in China, representing multiple brands. A total of 54 fertilizer samples were collected, consisting primarily of composts, including pig manure-based (*n* = 10), chicken manure-based (*n* = 12), cattle manure-based (*n* = 9), and sheep manure-based (*n* = 12) organic fertilizers, BOFs (*n* = 6), and “Other” organic fertilizers (*n* = 5). BOFs are a class of organic fertilizers that combine organic matter (OM) with viable functional microbial inoculants. This distinguishes them from conventional organic fertilizers, which contain no required viable microbes. The “Other” category comprised commercial products whose raw material composition was unknown, as the packaging was labeled only “organic fertilizer” and did not declare the feedstock source. All the commercial organic fertilizers were obtained from 15 provincial-level regions in China, including Beijing, Hebei, and Inner Mongolia (North China); Liaoning and Jilin (Northeast China); Gansu and Xinjiang (Northwest China); Anhui, Jiangsu, Jiangxi, and Shandong (East China); Henan and Hubei (Central China); and Guizhou and Yunnan (Southwest China). All products arrived as packaged, dry, free-flowing materials. On receipt, samples were homogenized by coning and quartering and stored at ambient temperature (18–25 °C) in light-protected, sealed containers for a maximum of seven days. Before storage, aliquots of each sample were flash-frozen and stored at −80 °C for subsequent antibiotic and ARG analysis and were not subjected to freeze–thaw cycles. Subsamples were prepared according to the requirement of each analyte. For pH, electrical conductivity, nutrient determination, antibiotic, ARB and ARG analyses, a subsample was ground to pass a 1 mm nylon sieve. For metal (Cu and Zn) determination, a subsample was ground to pass a 100-mesh (0.149 mm) sieve.

### 2.2. Measurements of Nutrient Content, Physicochemical Characteristics, and Heavy Metals

The nutrient content of commercial organic fertilizers was measured, including OM, total nitrogen (total N), total phosphorus (total P), available phosphorus (available P), and available potassium (available K). These parameters were determined using the potassium dichromate oxidation method, Kjeldahl method, molybdenum–antimony anti-spectrophotometric method, Olsen method (bicarbonate extraction), and flame photometric method (nitric acid dissolution), respectively. Electrical conductivity (EC) and pH were measured potentiometrically in a 1:5 (*w*/*v*) aqueous suspension (5 g sample in 25 mL deionized water) using a pH meter and EC meter, respectively. Concentrations of copper (Cu) and zinc (Zn) in organic fertilizers were quantified by digesting samples in HNO_3_:HClO_4_ = 5:1 (*v*:*v*) at 150 °C using a graphite digestion system (LabTech ED54, LabTech Instruments Co., Ltd., Beijing, China), followed by determination using inductively coupled plasma–optical emission spectrometry (ICP-OES) (PerkinElmer Optima 5300 DV, PerkinElmer, Inc., Waltham, MA, USA).

### 2.3. Determination of Antibiotics

The concentrations of 23 antibiotics—including 6 quinolones, 4 tetracyclines, 4 macrolides, and 9 sulfonamides (Table S1)—were determined. Antibiotic extraction and quantification followed the procedures described in the National Agriculture Industry Standard (Announcement No. 197-10-2019) [15], with method recovery rates of 70–120% and relative standard deviations (RSD) ≤ 20% as specified by the standard. The limits of detection (LODs) and limits of quantification (LOQs) for the target antibiotics ranged from 0.3 to 2 μg/kg and 1 to 5 μg/kg, respectively. Briefly, 2.0 g aliquots of each sample (with two replicates) were transferred into 50 mL centrifuge tubes. Subsequently, 3 mL of EDTA-McIlvaine buffer (Na_2_HPO_4_ 10.9 g/L, EDTA-Na_2_ 3 g/L, citric acid 12.9 g/L, pH 4.7) was added, followed by vortex mixing for 1 min. Precisely 10 mL of acetonitrile was added, and the mixture was immediately vortexed for 1 min. Thereafter, one QuEChERS salt extraction packet (Agilent Technologies, Santa Clara, CA, USA), containing 4 g of Na_2_SO_4_ and 1 g of NaCl was added and vortex-mixed for 1 min. The homogenized solutions were allowed to stand for 10 min to facilitate phase separation, then centrifuged at 9500 rpm for 10 min. From the resulting supernatant, an 8 mL aliquot was transferred to a 15 mL centrifuge tube and evaporated to near-dryness under a gentle nitrogen stream at 40 °C. The residue was reconstituted in precisely 2 mL of acetonitrile through vortex mixing. Concurrently, 0.5 g of lipid-removal/dispersive cleanup agent was activated in purification tubes by mixing with 2 mL deionized water. This activated sorbent was quantitatively transferred to the reconstituted sample solutions. Following 1 min of vortex mixing, purification was achieved through centrifugation at 9500 rpm for 5 min. Final supernatants were filtered through syringe membrane filters (0.22 µm) prior to introduction into the LC-MS/MS system.

Chromatographic separation was achieved using a C18 column (150 mm × 3.0 mm i.d., 1.8 µm particle size, or equivalent) maintained at 40 °C. A 2 µL injection volume was employed with a mobile phase flow rate of 0.4 mL/min, comprising (A) 0.2% (*v*/*v*) formic acid and 2 mmol/L ammonium acetate in aqueous solution and (B) 0.2% (*v*/*v*) formic acid in methanol, using the gradient elution program detailed in Table S2. Detection was performed using an HPLC system coupled to a SCIEX Triple Quad™ 5500 mass spectrometer (AB Sciex, Framingham, MA, USA).

### 2.4. Quantities of Total Culturable Bacteria and Resistant Bacteria

The total culturable bacteria (TCB) were enumerated on non-selective beef extract peptone agar, whereas the oxytetracycline (OTC)-resistant, sulfamethoxazole (SMX)-resistant, and dual (OTC and SMX)-resistant bacteria were enumerated on the same base medium amended with the corresponding antibiotic(s). OTC and SMX were selected as representative antibiotics of the tetracycline and sulfonamide classes, respectively, which are among the most extensively used veterinary antibiotics in Chinese livestock production [16,17]. The screening concentrations were 16 mg/L for OTC and 76 mg/L for SMX, corresponding to the clinical resistance breakpoints established by the Clinical and Laboratory Standards Institute (CLSI M100-S20) [18]. Specifically, 16 mg/L is the resistance breakpoint for the tetracycline class, and 76 mg/L is the sulfamethoxazole component of the trimethoprim–sulfamethoxazole (1:19) resistance breakpoint (R ≥ 4/76 μg/mL) (CLSI M100-S20). These concentrations have been previously applied for ARB screening in swine manure and composting studies [19].

A beef extract–peptone agar medium (5 g/L beef extract, 10 g/L peptone, 5 g/L NaCl, 20 g/L agar) was prepared in distilled water, adjusted to pH 7.2, and autoclaved at 121 °C for 20 min. When cooled to approximately 45 °C, filter-sterilized OTC and/or SMX (dissolved in methanol) were aseptically added to achieve final concentrations of 16 mg/L and 76 mg/L, respectively. Solvent-only control plates were included for each batch. These plates contained the base medium plus an equal volume of methanol but no antibiotic, confirming that the methanol carrier did not inhibit growth. Then, 2.00 g of fresh organic fertilizer samples were precisely weighed and transferred into 18 mL of sterile physiological saline (0.85% NaCl). The mixtures were oscillated at 200 rpm for 30 min in a shaker to achieve complete homogenization (yielding a 10^−1^ dilution). Serial dilutions were then prepared up to 10^−7^. For each sample–dilution combination, triplicate plates were prepared, and 100 µL of the appropriate dilution (yielding 30–300 colonies) was spread onto pre-prepared agar plates. After inverted incubation at 37 °C for 48 h, colonies on plates with 30–300 counts were enumerated as colony-forming units (CFUs). Colony counts were converted to log_10_ CFU per gram of sample weight. The proportion of antibiotic-resistant bacteria relative to the TCB count was also calculated to account for differences in total bacterial abundance among fertilizer types.

### 2.5. DNA Extraction and Quantification of Gene Elements

Total microbial genomic DNA was extracted using the OMEGA Mag-Bind Soil DNA Kit (M5635-02) (Omega Bio-Tek, Norcross, GA, USA), following the manufacturer’s instructions, and stored at −20 °C prior to further analysis. The 16S rRNA gene (as a marker for total bacterial abundance), ARGs, and the class 1 integron gene (intI1) in each sample were quantified using a quantitative real-time PCR (qPCR) assay. The target genes included four tetracycline resistance genes (*tet* genes, *tetG*, *tetL*, *tetW* and *tetX*), two sulfonamide resistance genes (*sul* genes, *sul1* and *sul2*), two chloramphenicol resistance genes (*cml* genes, *cmlA* and *floR*), and three resistance genes grouped as *mla* genes—the macrolide resistance gene *ermB*, the lincosamide resistance gene *lnuA*, and the aminoglycoside resistance gene *aadA*—together with *intI1*, a proxy for the potential horizontal dissemination of ARGs. The 16S rRNA gene was quantified using primers described by Suzuki et al. [20]. The sequences of qPCR primers for all target genes are listed in Table S3. The 20 µL reaction mixtures consisted of 10 µL of 2× SYBR Green real-time PCR premix (Tiangen, Beijing, China), 0.2 µM of each primer, and 1 µL of template DNA. qPCR amplifications were performed under the following conditions: an initial denaturation step of 5 min at 95 °C, followed by 40 cycles of denaturation at 95 °C for 15 s, annealing at the gene-specific optimized temperature for 30 s, and extension at 72 °C for 30 s. The threshold cycle (Ct) was used to estimate the initial DNA copy number. The absolute abundance of ARGs was expressed as copies per gram dw. Additionally, the normalized abundance of each ARG was calculated as the ratio of its copy number to that of the 16S rRNA gene (ARG copies/16S rRNA gene copies) to enable comparison of ARG prevalence across samples independent of total bacterial abundance.

### 2.6. Data Analysis

All data were analyzed and visualized using R 4.5.2 software [21]. The Kruskal–Wallis (K-W) test was used to assess statistical differences among groups using the rstatix package (v0.7.2), and Dunn’s test was conducted for post hoc pairwise comparisons with the Benjamini–Hochberg method for *p*-value adjustment in the FSA package (v0.10.1). Results were visualized using the ggplot2 package (v3.5.2) [22]. Associations between physicochemical indicators and ARB were assessed by Spearman’s rank correlation, analyzed separately for absolute log10 counts and TCB-normalized ARB proportions. Because each framework comprised 40 pairwise tests, Benjamini–Hochberg false-discovery-rate correction (α = 0.05) was applied; heatmap cells show ρ and significance asterisks from unadjusted *p* values, whereas FDR-adjusted *p* values are listed in Table S4 within the Supplementary Materials. Additionally, redundancy analysis (RDA) was performed to examine the relationship between ARG profiles and environmental factors using the vegan package [23]. Prior to RDA, ARG abundance data were log10(x + 1)-transformed to normalize distributions and reduce the influence of extreme values. Individual antibiotic concentrations were summed to obtain a total antibiotic concentration, which was then included in the RDA as a single environmental variable. To address multicollinearity, variance inflation factors (VIFs) were calculated for all candidate predictors via the vif.cca function; variables with VIF < 20 were retained in the final RDA model [24]. Significance testing of constrained axes and individual variables was conducted through permutation-based ANOVA (999 permutations).

To compare the contributions of fertilizer type and geographic origin to variation in fertilizer properties, permutational multivariate analysis of variance (PERMANOVA) was performed with the adonis2 function (vegan package, R), fitting fertilizer type and geographic region as fixed factors and using Type III (marginal) sums of squares (by = “margin”; 999 permutations) so that the effect of region was evaluated after statistically controlling for fertilizer type (i.e., region|type). For each of the four indicator groups (physicochemical properties, contaminants, antibiotic-resistant bacteria, and ARGs), variables were standardized by z-score and Euclidean distances computed; R^2^ values were used to quantify and compare the effect sizes of the two factors. Because fertilizer type and geographic region are partly confounded in this observational design, an additional within-type PERMANOVA was performed: for each fertilizer type, adonis2(distance ~ region) tested, separately, whether region explained significant additional variation once type was held constant. Prior to each PERMANOVA, homogeneity of multivariate group dispersions was verified with betadisper, and significance was assessed at α = 0.05.

## 3. Results

### 3.1. Nutrients and Physicochemical Characteristics in Organic Fertilizers

**Figure 1:**
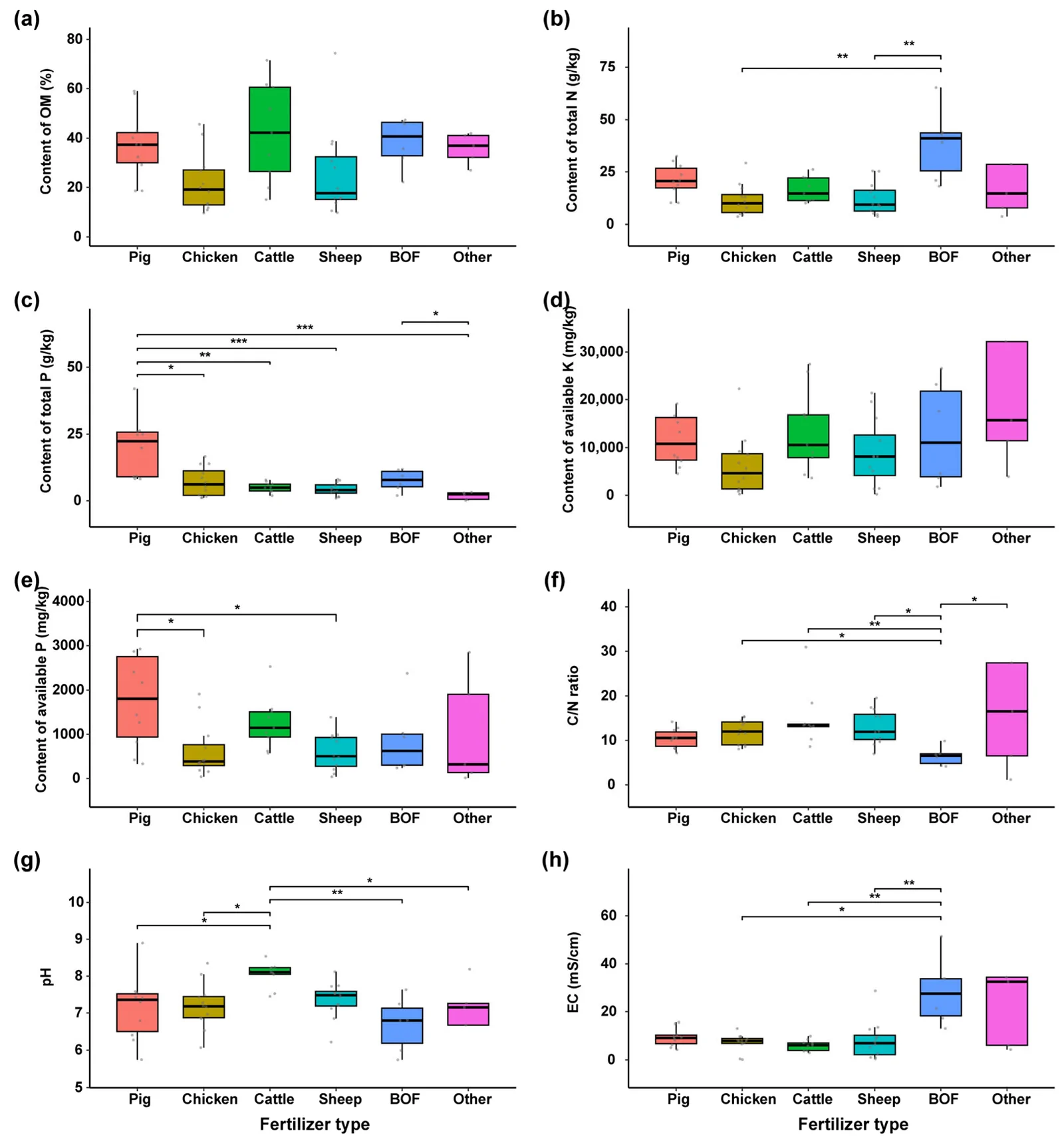
Nutrient content and physicochemical parameters in commercial organic fertilizers by fertilizer type. (**a**) OM; (**b**) total N; (**c**) total P; (**d**) available K; (**e**) available P; (**f**) C/N ratio; (**g**) pH; (**h**) EC. * *p* < 0.05, ** *p* < 0.01, *** *p* < 0.001.

Soil organic matter (OM) and the key plant nutrients total N, total P, available P, and available K were determined in this study. Based on fertilizer types, the median OM and available K were comparable among different fertilizers (Figure 1a,d). The total N was significantly higher in BOF than in chicken and sheep manure-based fertilizers (*p* < 0.05; Figure 1b). The total P in pig manure-based fertilizer was significantly higher than that in chicken, cattle, sheep manure-based, and other fertilizers (*p* < 0.05, Figure 1c). The content of available P was significantly higher than in chicken and sheep manure-based fertilizer (*p* < 0.05, Figure 1e). Of the 54 organic fertilizer samples, 26 samples (48.15%) had OM content below the 30% threshold specified in China’s organic fertilizer industry standard NY/T 525-2021 [25]. The C/N ratio was highest in BOF and significantly higher than that of all other fertilizers (*p* < 0.05, Figure 1f) except pig manure-based fertilizer (*p* > 0.05). According to the California Compost Quality Council (CCQC) [26], the maximum accepted C/N ratio of the finished composts is 20. Only three C/N ratio values exceeded 20 (two from “Other” category and one from cattle manure-based fertilizer). Additionally, imbalanced N–P_2_O_5_ ratios (<2:1) were observed in 77.8% of organic fertilizer samples. In particular, all pig manure-based samples fell below this threshold, with ratios significantly lower than those in cattle and sheep manure-based fertilizers, BOF, and other types.

pH and EC were determined as the physicochemical characteristics of organic fertilizers. The results showed that cattle manure-based fertilizer had the highest pH value, which was significantly higher than that of chicken manure-based and BOF fertilizers (Figure 1g). EC was relatively high in most (45/54, 83.33%) of the organic fertilizers (>4 mS/cm), and EC in BOF was significantly higher than that in chicken, cattle, and sheep manure-based fertilizers (Figure 1h). According to the standard NY/T 525-2021 [25], pH should fall within the range of 5.5–8.5; the majority of organic fertilizers (94.44%) met this standard. The upper limit of EC set by the California Compost Quality Council [26] and British Standards PAS-100 [27] for compost used in agricultural applications is 4 dS/m (4000 µS/cm) [28], and 100% of organic fertilizers met this standard.

In the region-grouped comparisons (Figure S1a–h), organic fertilizers from Southwest China contained the highest OM, whereas those from Northeast China tended to be lower in OM, total N, available K, and available P. Total P was comparable across origins (Figure S1c). pH and EC were generally comparable among origins, except that Northeast China had significantly lower levels than North and Northwest China (Figure S1g,h). These regional patterns are descriptive and partly confounded by fertilizer type and processing. A two-factor PERMANOVA showed that fertilizer type, not region, was the dominant driver of physicochemical variation (type R^2^ = 0.22, *p* = 0.001 vs. region R^2^ = 0.15, *p* = 0.005), and within-type analyses indicated that region explained little additional variance, except with regard to sheep-derived fertilizers (Table S5).

### 3.2. Heavy Metals and Antibiotics in Organic Fertilizers

**Figure 2:**
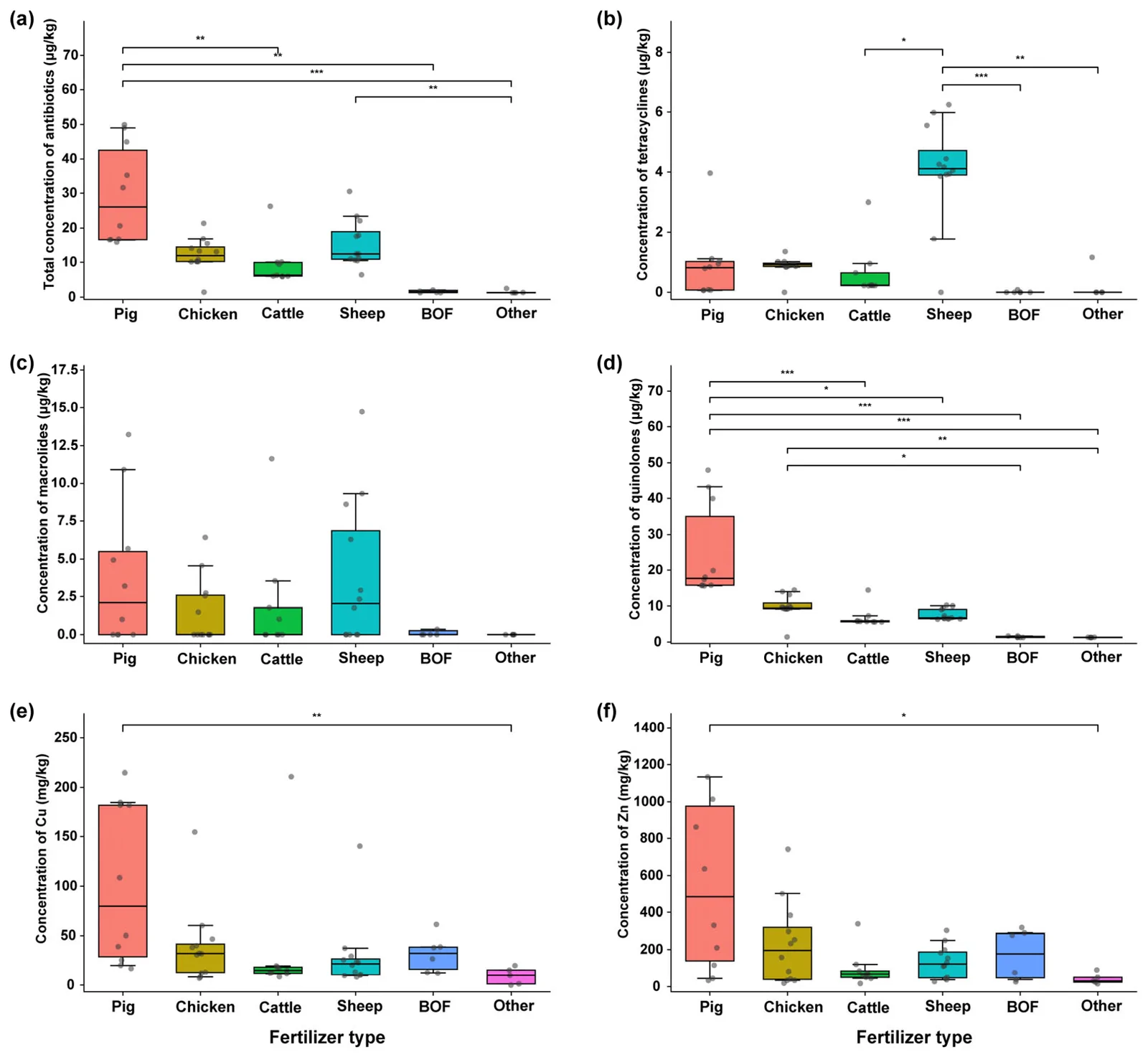
Concentration of antibiotics and heavy metals in commercial organic fertilizers. (**a**) Total antibiotics; (**b**) tetracyclines; (**c**) macrolides; (**d**) quinolones; (**e**) Cu; (**f**) Zn. * *p* < 0.05, ** *p* < 0.01, *** *p* < 0.001.

The total concentration of the measured antibiotics ranged from 1.27 to 49.62 µg/kg dw across all organic fertilizers. Pig manure-based fertilizers (median 26.12 µg/kg) contained higher antibiotic concentrations than cattle manure-based (median 6.25 µg/kg), BOF (median 1.37 µg/kg), and ‘Other’ (median 1.28 µg/kg) fertilizers (Figure 2a). Sheep manure-based fertilizers contained a higher concentration of tetracyclines than cattle manure-based, BOF, and ‘Other’ fertilizers (Figure 2b). There was no significant difference in macrolide concentrations among fertilizer types (Figure 2c), whereas quinolone concentrations were highest in pig manure-based fertilizers (median 17.70 µg/kg) and were significantly higher than those in all other types except chicken manure-based fertilizers (median 9.45 µg/kg). Quinolones accounted for the majority of the total antibiotic concentration in pig manure-based fertilizers, at concentrations an order of magnitude higher than those of tetracyclines. Table S1 lists the detection frequency of each antibiotic class; quinolones were the most frequently detected, whereas none of the nine sulfonamides were detected in any sample. By region, no significant difference in the total concentration of the 23 measured antibiotics was observed (Figure S2a).

Cu and Zn concentrations showed the greatest variation among pig manure-based fertilizers. Cu ranged from 16.71 to 214.84 mg/kg dw and Zn from 33.43 to 2175.79 mg/kg dw in pig manure-based fertilizers. These concentrations were significantly higher than those in the “Other” category of organic fertilizers. Since China’s current organic fertilizer standard NY/T 525-2021 [25] does not specify limits for Cu and Zn, German limit standards [29] were used for reference. Based on the German limit standards for Cu (100 mg/kg) and Zn (400 mg/kg) in manure compost, 50% of pig, 8.33% of chicken, 11.11% of cattle, and 8.33% of sheep manure-based fertilizers exceeded the Cu limit. For Zn, 50% of pig and 16.67% of chicken manure-based fertilizers exceeded the limit. Overall, the exceedance rates of Cu and Zn across all organic fertilizers were 14.81% and 12.96%, respectively. By region, Cu and Zn concentrations showed no significant differences (Figure S2e,f).

### 3.3. Enumeration of Antibiotic-Resistant Bacteria in Organic Fertilizers

**Figure 3:**
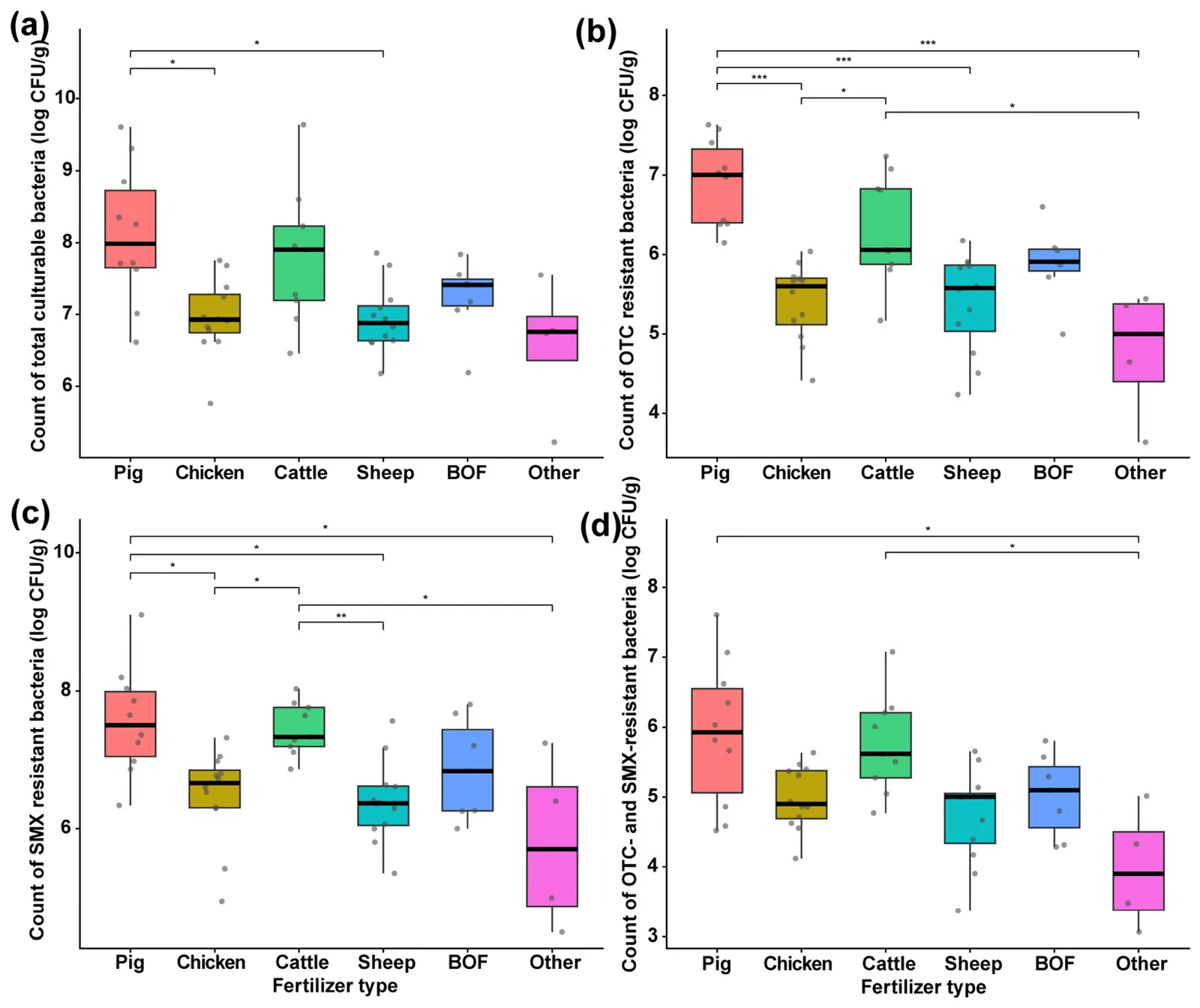
Count of total culturable bacteria (TCB) and antibiotic-resistant bacteria (ARB) resistant to oxytetracycline (OTC), sulfamethoxazole (SMX), and dual-resistant (OTC and SMX) in commercial organic fertilizers. (**a**) TCB; (**b**) OTC-resistant bacteria; (**c**) SMX-resistant bacteria; (**d**) OTC- and SMX-resistant bacteria. * *p* < 0.05, ** *p* < 0.01, *** *p* < 0.001.

The TCB counts ranged from 1.69 × 10^5^ to 4.32 × 10^9^ CFU/g dw. Pig manure-based fertilizers showed significantly higher TCB counts than those in chicken- and sheep manure-based fertilizers (*p* < 0.05, Figure 3a), whereas no significant difference was detected among other types of fertilizers. For OTC-resistant bacteria, pig manure-based fertilizers had significantly higher counts than those in chicken-, sheep-, and ‘Other’ organic fertilizers (*p* < 0.001, Figure 3b), and cattle manure-based fertilizers had significantly higher counts than those in chicken- and ‘Other’ organic fertilizers (*p* < 0.05). For SMX-resistant bacteria, both pig and cattle manure-based fertilizers had significantly higher counts than those in chicken-, sheep-, and “Other” organic fertilizers (*p* < 0.05, Figure 3c). For dual-resistant (OTC and SMX) bacteria, pig and cattle manure-based fertilizers had significantly higher counts than “Other” organic fertilizers (*p* < 0.05, Figure 3d).

To account for the inherently higher total bacterial abundance in pig and cattle manure-based fertilizers, the relative proportion of ARB to TCB was also calculated. The results showed that the ratios of OTC-, SMX-, and OTC- and SMX-resistant bacteria to TCB did not differ significantly among fertilizer types (Kruskal–Wallis, *p* > 0.05; Figure S4). Geographically, both the count of ARB and the relative proportions of ARB to TCB were generally comparable among origins (Figures S3 and S4).

Spearman correlation was used to examine associations between physicochemical properties and ARB counts, aiming to identify potential drivers of ARB abundance. At the absolute-count level (Figure S5a), total P, Cu, and available P showed significant moderate positive correlations with both OTC-resistant and dual-resistant bacteria (0.3 < ρ < 0.5, *p* < 0.05). Total N and OM were also significantly correlated with OTC-resistant bacteria and Zn and antibiotics with dual-resistant bacteria. For TCB, OM and total P showed significant correlations of moderate (ρ = 0.33) and weak (ρ = 0.27) magnitude, respectively (both *p* < 0.05). By contrast, after normalizing ARB counts to TCB (ARB/TCB; Figure S5b), only available P remained significantly positively correlated with OTC-resistant bacteria (ρ = 0.36, *p* < 0.01). No other indicator showed a significant association with the normalized ARB proportions.

### 3.4. Antibiotic Resistance Genes in Organic Fertilizers

**Figure 4:**
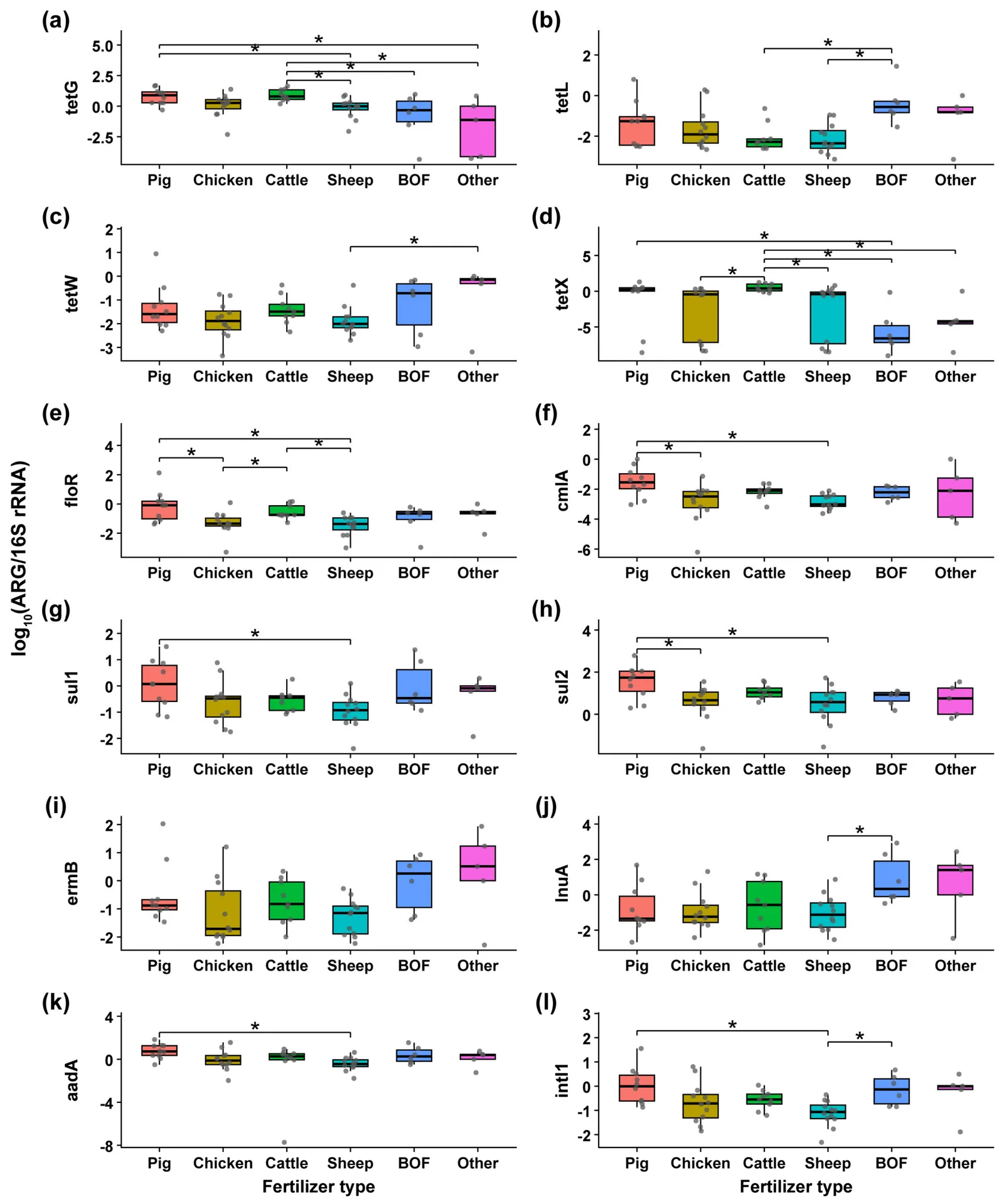
Abundance of antibiotic resistance genes (ARGs) and the integron gene *intI1* in commercial organic fertilizers. (**a**) *tetG*; (**b**) *tetL*; (**c**) *tetW*; (**d**) *tetX*; (**e**) *floR*; (**f**) *cmlA*; (**g**) *sul1*; (**h**) *sul2*; (**i**) *ermB*; (**j**) *lnuA*; (**k**) *aadA*; (**l**) *intI1*. Kruskal–Wallis test with Dunn’s * *p* < 0.05.

The absolute abundances of 11 ARGs and of the class 1 integron gene *intI1* were quantified by qPCR. The absolute abundance of most ARGs was highest in pig manure-based fertilizer, followed by cattle manure-based fertilizer, whereas chicken and sheep manure-based fertilizers and BOF generally contained lower ARG levels (Figure 4). The target *tet* genes (*tetG*, *tetL*, *tetW* and *tetX*), sul genes (*sul1* and *sul2*), the macrolide resistance gene *ermB*, the aminoglycoside resistance gene *aadA*, and *intI1* (a proxy for the potential horizontal dissemination of ARGs) all showed elevated levels in pig and cattle manure-based fertilizers. Geographically, the absolute abundance of most ARGs did not differ significantly among regions across China (Figure S6).

The 16S rRNA gene abundance was also determined to enable normalization of the ARG data (Figure S7). Consistent with the ARG results, the absolute abundance of the 16S rRNA gene was also highest in pig and cattle manure-based fertilizers. After normalization to 16S rRNA gene abundance, the pattern shifted mainly toward pig manure-based fertilizer. Eight target genes (*tetG*, *tetX*, *floR*, *cmlA*, *sul1*, *sul2*, *aadA*, and *intI1*) remained highest in pig manure-based fertilizer, and their normalized abundances were significantly greater than those in at least one other fertilizer category. Only three target genes (*tetG*, *tetW*, and *floR*) continued to show relatively higher normalized abundances in cattle manure-based fertilizer, also differing significantly from at least one other fertilizer type. After normalization to 16S rRNA gene abundance, the pattern shifted mainly toward pig manure. Eight target genes (*tetG*, *tetX*, *floR*, *cmlA*, *sul1*, *sul2*, *aadA* and *intI1*) remained highest in pig manure-based fertilizer, and their normalized abundances were significantly greater than those in at least one other fertilizer category. Only three target genes (*tetG*, *tetW* and *floR*) showed relatively higher normalized abundances in cattle manure-based fertilizer than the other categories excluding pig manure. Each of these three genes also differed significantly from at least one other fertilizer type (Figure S8). By region, none of the ARGs showed a significant difference in normalized abundance among regions across China (Figure S9).

### 3.5. Redundancy Analysis of Antibiotic Resistance Genes and Physicochemical Characteristics

The RDA including ten physicochemical variables (OM, total N, total P, available K, available P, pH, EC, Cu, Zn, and antibiotics) explained 57.03% of the total variation in ARG composition (Figure S10). Forward selection identified six variables (total N, total P, available K, pH, EC, and Cu) that contributed significantly; the reduced six-factor model explained 43% of the variation (R^2^adj = 0.43, F = 7.04, *p* = 0.014, 999 permutations). Among these, available K (R^2^ = 0.21, *p* = 0.014), pH (R^2^ = 0.21, *p* = 0.005), and EC (R^2^ = 0.26, *p* = 0.009) showed the largest marginal effects. These results indicate that the six significant environmental drivers are collectively associated with the structure of ARG composition. In contrast, antibiotics showed no significant contribution to ARG composition in the RDA (*p* > 0.05).

### 3.6. Comprehensive Quality Assessment of Commercial Organic Fertilizers

**Figure 5:**
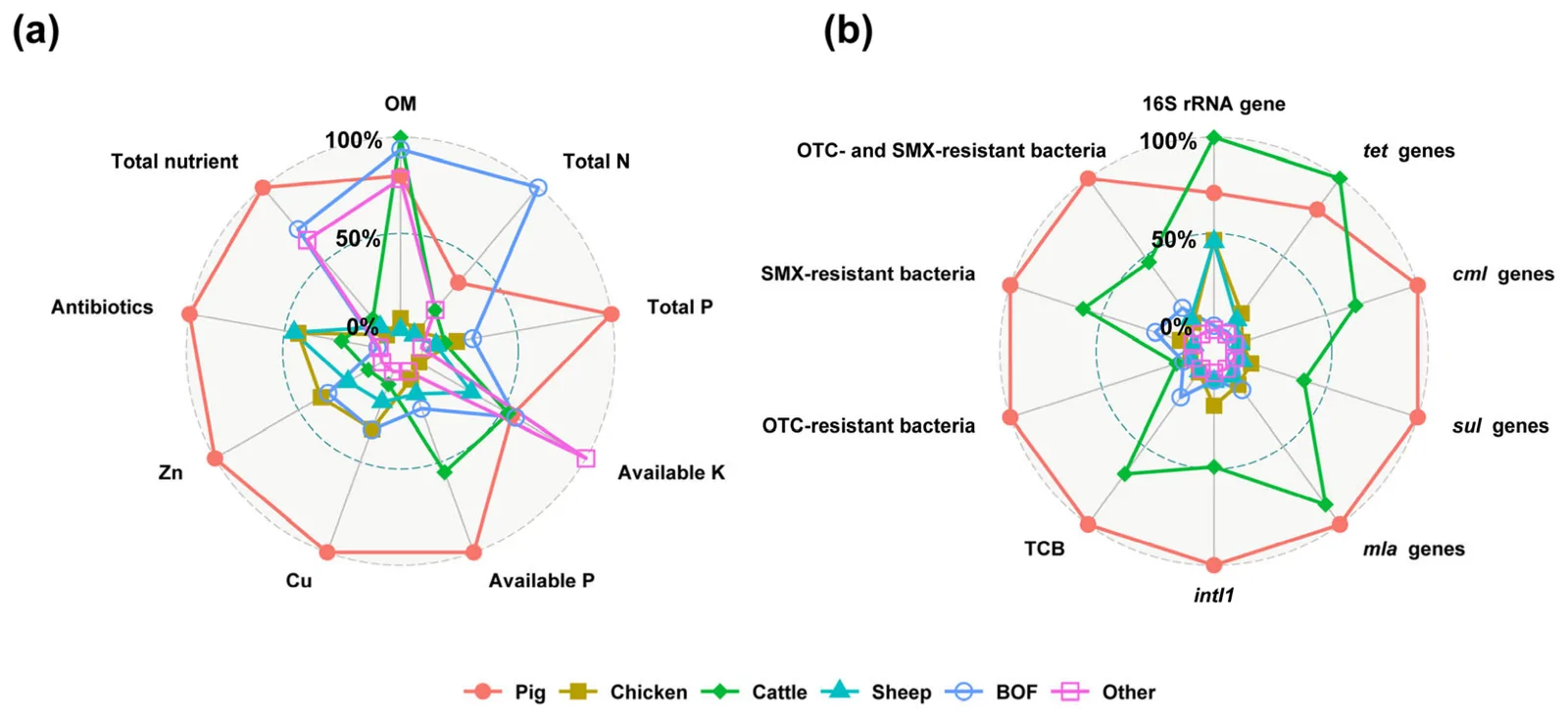
Comprehensive profiles of commercial organic fertilizers. (**a**) Nutrient and chemical pollutant indicators; (**b**) microbiological parameters, including culturable bacterial counts and gene abundance. Standardization method: All indicators were min–max normalized to [0, 1] scale.

To evaluate organic fertilizer quality, composite radar plots were constructed by combining median nutrient and chemical pollutant indicators (Figure 5a) and, separately, median culturable bacterial counts with ARG abundance (Figure 5b). The plots showed that pig manure-based fertilizer, although richer in nutrients than the chicken, cattle, and sheep manure-based fertilizers, also had the highest levels of both chemical and microbial contaminants. Cattle manure-based fertilizer had high OM and low chemical contaminants but ranked second in ARB and ARG abundance after pig manure-based fertilizer. Chicken and sheep manure-based fertilizers had low levels of both nutrients and contaminants. In contrast, BOF combined high nutrient levels with low contaminant concentrations and, specifically, the highest contents of OM, total N, and total P (Figure 5a). Therefore, when nutrient, chemical, and microbial parameters were integrated, BOF could be considered the optimal organic fertilizer among those evaluated.

Overall, fertilizer type accounted for more of the multivariate variation than geographic region in all four indicator groups (physicochemical properties including nutrients, antibiotics and heavy metals, ARB counts, and ARG abundances), with PERMANOVA R^2^: 0.20–0.38 versus 0.07–0.15. Type effects were significant in every group (all *p* ≤ 0.003), but region reached significance only for physicochemical properties (*p* = 0.005) and was non-significant for the other three (Figure S11).

## 4. Discussion

The application of organic fertilizers can help improve soil organic matter and soil structure [30]. Among the 54 organic fertilizer samples collected in this study, 48.15% had OM content below the threshold stipulated by China’s current organic fertilizer industry standard NY/T 525-2021 [25]. It has been reported that low OM content (<30%) usually indicates that organic fertilizer has been mixed with sand or soil [31], which may partly account for our findings. This result suggests that stricter quality control measures should be implemented for organic fertilizers to ensure compliance with nutrient content standards. BOF contained the highest total N and the lowest C/N, indicating both a greater N pool and faster mineralization relative to chicken- and sheep manure-based products, which suggests a higher readily available N supply capacity (Figure 1b,f). Additionally, Roberts and Majumdar [32] suggested an ideal N–P_2_O_5_ ratio of 2:1, while another study recommended an ideal N–P_2_O_5_ ratio of 4.4:1–8.7:1, due to the considerable variance in actual plant and ecosystem requirements [33]. However, 77.8% of the organic fertilizers investigated in this study exhibited N–P_2_O_5_ ratios below 2:1, and 57.4% were even below 5:4. Such imbalanced nutrient ratios suggest that applying organic fertilizer based solely on N requirements may lead to excessive P input, creating a risk of P accumulation in soil and associated eutrophication of surface waters [34]. Therefore, integrated measures should be taken to avoid potential eutrophication, such as adjusting application rates based on soil nutrient status and P content in specific organic fertilizers, and supplementing N from other sources.

EC and pH are among the commonly tested chemical characteristics of composts. The pH of compost can influence soil pH and microbial communities [30]. Previous studies have found that pH is not a key factor limiting composting, as most materials fall within the pH range of 5.5–8.0 [35]. Our results are consistent with these findings, as 94.44% of the compost samples had pH values within this range, reflecting that pH was largely acceptable. EC reflects the soluble ion concentration and salinity of com-post and correlates with microbial mineralization, nutrient availability, and phytotoxicity [36,37]. Application of composts with high EC may increase soil EC and negatively affect plant growth [28]. In the present study, only 16.67% of the commercial organic fertilizers fell below the CCQC/PAS-100 EC benchmark of 4 mS/cm (4 dS/m), indicating that salt was the weak point in current commercial organic fertilizers, and further work therefore is needed to reduce the soluble salt content.

Antibiotics are commonly used feed additives in animal husbandry, and the pig industry generally uses more antibiotics and heavy metals than other livestock sectors [17]. This is largely because intensive, high-density pig production and early weaning elevate disease pressure (e.g., post-weaning diarrhea), increasing the reliance on antibiotics for prophylaxis, treatment, and growth promotion [38]. This also leads to higher concentrations of antibiotics and heavy metals in pig manure [18]. In our study, quinolones had both the highest concentration and detection frequency among the measured antibiotics, followed by tetracyclines and macrolides. This result is consistent with a previous study conducted in China that reported quinolones were detected in 100% of manure-based fertilizer samples, followed by tetracyclines [19]. In general, tetracyclines are the most widely used antibiotics in the livestock industry [16,39] and are frequently the most concentrated antibiotic class in animal manure compared to other classes [17,40]. However, unlike fresh animal manure, commercial manure-based organic fertilizers have typically undergone composting and storage for an unknown period before marketing, during which the fate of different antibiotic classes varies. The prevalence of quinolones in these composted products corroborates the previous conclusion that quinolones exhibit high chemical stability and are resistant to degradation under increased temperature or hydrolysis [41]. This resistance can be attributed to their C–F bonds, which are difficult to cleave [42].

Heavy metals are also frequently added to animal feed in addition to antibiotics, particularly Cu and Zn [43]. These elements are routinely supplemented at supra-nutritional (or pharmacological) levels, primarily to improve health and growth performance and to prevent enteric diseases such as diarrhea, benefits that are largely attributed to their antimicrobial activity against gut pathogens [44,45]. The Cu content in most (79.6%) organic fertilizers was below 50 mg/kg, with two samples exceeding 200 mg/kg (one pig and one cattle manure-based fertilizer) (Figure 2e). The Zn content in organic fertilizers was generally below 500 mg/kg (87.0%), with two pig manure-based samples exceeding 1000 mg/kg (Figure 2f). Based on German limit standards [29], we found that 50% of pig manure-based fertilizers exceeded German limits for Cu (100 mg/kg) and Zn (400 mg/kg). These frequencies were comparable to those reported in an investigation conducted in Ningxia, China, which found exceedance rates of 50% for Cu and 66.7% for Zn [14]. Overall, the exceedance rates of Cu and Zn across all organic fertilizers in this study were 14.81% and 12.96%, respectively. These rates were lower than the 35.0% reported for Cu but comparable to the 13.3% reported for Zn in another study investigating heavy metals in organic fertilizers across China [13].

Standard plate count on antibiotic-supplemented media is a common approach to assessing the phenotypic resistance of bacterial communities. OTC and SMX are both commonly used antibiotics in animal husbandry and serve as representative compounds for investigating bacterial phenotypic resistance. Using this method, we found that the counts of TCB, OTC-resistant, and dual-resistant bacteria were all highest in pig manure-based fertilizers, followed by cattle manure-based fertilizers (Figure 3). The inter-type variation in TCB itself is noteworthy. At the absolute level, TCB showed nominal positive associations with organic matter (ρ = 0.33, *p* = 0.014) and total phosphorus (ρ = 0.27, *p* = 0.046; Figure S5a), but neither survived Benjamini–Hochberg correction and both were modest; pH, EC, Cu, and Zn showed no significant association with TCB. These nutrient–TCB links largely reflect that manure-based fertilizers—especially pig and cattle—are simultaneously microbially denser and richer in OM and P, rather than indicating that a single chemical property independently drives the culturable load. The differences therefore most plausibly originate from the fertilizer feedstock: pig and cattle manures intrinsically harbor denser microbial communities than poultry, sheep, or non-manure-based (BOF/other) substrates, and this source-derived microbial load is retained through composting and drying [46,47]. When expressed as relative proportions of TCB, there was no longer significant difference between fertilizer types (Figure S4). This suggests that the observed differences in absolute ARB abundance were primarily attributable to variations in total bacterial load rather than specific enrichment of resistance.

Our correlation analysis showed that, at the absolute count level, total P, available P, OM, Cu, Zn, and antibiotic residues were all positively correlated with ARB counts or TCB (ρ = 0.27–0.49, *p* < 0.05; Figure S5a), yet only the nutrient–OTC pairs survived Benjamini–Hochberg correction, whereas the metal and antibiotic associations did not. These linkages reflect co-occurrence of these physicochemical factors with overall microbial biomass (TCB), rather than resistance selection. They largely vanished once ARB were normalized to TCB (Figure S5b): only available P retained an association with OTC-resistant bacteria, and even that did not survive correction (adjusted *p* ≈ 0.28). Absolute counts do reflect the actual resistance risk and retain real exposure value, but the survey of commercial organic fertilizers gave no evidence that any measured physicochemical factor, including contaminants, drives ARB dissemination. The normalized proportions showed no consistent strong association with resistant bacteria, so the absolute-count patterns could not be attributed to specific selective drivers. Although previous studies advocated prioritizing control of heavy metal and antibiotic co-contamination to curb ARB dissemination [43], our lack of clear signals most likely reflects limited sample size and the substantial heterogeneity among the surveyed fertilizers, which tempers that emphasis.

In this study, qPCR was also used to investigate genotypic resistance in commercial organic fertilizers. The results were similar to those of the phenotypic resistance assessment, showing that bacterial resistance in pig and cattle manure-based fertilizers was greater than in other types of compost fertilizers. The comparison of ARGs between chicken and cattle manure-based fertilizers was not consistent with a previous study, which reported higher absolute ARG abundance in chicken manure than in cattle manure [14]. This discrepancy may be attributed to the substantial differences in pollutant content and physicochemical characteristics between the two studies; moreover, their samples were fresh animal feces rather than composted fertilizers. For example, tetracycline concentrations in their chicken and pig feces (7148.8–206,477.2 µg/kg) were much higher than those in cattle and sheep feces (143.7–626.5 µg/kg), which likely induced higher ARG abundance in chicken than in cattle feces. Additionally, the composting process results in different elimination rates of specific ARGs in manure derived from different animal species [48].

The RDA indicated that six material physicochemical variables (total N, total P, available K, pH, EC, and Cu) were most strongly associated with variation in the ARG profiles, collectively explaining the largest proportion of resistome structuring across fertilizer types. Notably, although antibiotics are recognized inducers of ARG expression, they showed no significant correlation with ARG profiles in the RDA. This may be because antibiotic residues in matured commercial organic fertilizers are generally low (<100 µg/kg) and therefore affect ARG profiles less strongly than heavy metals such as Cu or other chemical variables. Moreover, many antibiotics are prone to degradation, which diminishes or eliminates their induction of ARGs, whereas heavy metals resist degradation. Consequently, the persistent co-selective pressure exerted by heavy metals on ARGs leaves a lasting impact on ARG profiles [49]. In addition, the elevated *intI1* abundance observed in pig and cattle manure-based fertilizers suggests an increased potential for HGT in these fertilizer types. These findings suggest that managing heavy metals and certain chemical variables in commercial organic fertilizers should generally be prioritized over antibiotic control, because these physicochemical factors exert a stronger impact on ARG profiles than antibiotics.

Fertilizer type explained a greater proportion of the variation in all four indicator groups (Figure S11). This mainly reflects differences in animal source and husbandry practices rather than collection province. Livestock manure-based organic fertilizers generally exhibited higher contamination levels than those in the “Other” category. Pig and cattle manure-based fertilizers were particularly elevated. This indicates that the manure substrate, rather than geographic origin, is the main determinant of resistance loading in commercial fertilizers. For veterinary antibiotics, the observed ranking of manure contamination, with pig and cattle highest, is consistent with the global usage gradients reported in [50], which showed that antibiotic quantities per unit biomass decreased in the order pigs (173.1 mg PCU^−1^) > cattle (59.6 mg PCU^−1^) > chickens (35.4 mg PCU^−1^). Although China does not publish a species-split national antibiotic database, this gradient is highly relevant here because China and the United States together account for nearly 60% of the global antimicrobial footprint [51], with China by far the larger user (≈41.0 kt in 2020 versus ≈7.0 kt for the United States). For Cu and Zn, the elevated concentrations in pig-based fertilizer reflect species-specific feeding practices. As trace elements, Cu and Zn are routinely supplemented to livestock feeds, with particularly high additions to pig diets. Pigs are far less sensitive to Cu toxicity than ruminants (sheep and lambs tolerate only 20 and 100 mg/kg, respectively, whereas pigs routinely receive growth-promoting Cu at 75–250 mg/kg diet) [52]. Because Cu and Zn are poorly absorbed and largely excreted [53], this higher feed input is transferred directly into higher manure concentrations, explaining why pig manure carried the greatest Cu and Zn loads.

The type effect was most pronounced for contaminants (R^2^ = 0.38), pointing to large differences in heavy metal and veterinary antibiotic burdens across manure types (Figure S11). In contrast, geographic region was significant only in terms of physicochemical properties, where its explanatory power (R^2^ = 0.15) was lower than that of fertilizer type (R^2^ = 0.22) yet still substantial. For the other three groups, the regional effect was negligible. For ARB and ARGs, the negligible regional signal indicates that resistance traits are a manure type-specific issue rather than a geographically pervasive contamination, implying that regulating fertilizers by animal source category would be more effective than region-based controls.

## 5. Conclusions

This study sampled 54 commercial organic fertilizers across China, spanning the main fertilizer types and geographic origins, to provide a comprehensive assessment of nutrient content, chemical contaminants, ARB, and ARGs. The results showed that nutrient concentrations differed markedly across fertilizer types and that ARB and ARGs were most abundant in pig manure. Antibiotic concentrations were generally low. However, Cu and Zn showed non-negligible exceedance rates relative to the standards (14.81% and 12.96%, respectively); 83.33% of EC values exceeded the limit of 4 mS/cm, warranting stricter control of heavy metals and soluble salts in commercial organic fertilizers. Overall, fertilizer type drove most of the variation in nutrients and contaminants, more than geographic origin did. Among animal manure-based fertilizers, pig manure had the highest nutrient and contaminant levels, followed by cattle manure. Chicken and sheep manure showed lower environmental risk but lower nutrient concentrations than pig and cattle manure. By contrast, BOF combined elevated nutrient content with markedly lower contaminant levels, making it a preferable sustainable alternative to animal manure-based fertilizers. This study enhances our understanding of the prevailing quality of commercial organic fertilizers in China and provides an evidence base for controlling contaminants in these agricultural inputs. Future work is needed for managing nutrients and controlling pollutants at the same time through source- and species-specific regulation.

## Data Availability

The original contributions presented in this study are included in the article and the Supplementary Material (Supplementary_Data_S1.xlsx). Further inquiries can be directed to the corresponding author.

## Supplementary Materials

The following supporting information can be downloaded at https://www.mdpi.com/article/10.3390/microorganisms14102364/s1, Table S1: Detection frequency (%) of antibiotics in different types of organic fertilizers; Table S2: Gradient elution program; Table S3: Primer sets used in this study; Table S4: Spearman correlations between fertilizer indicators and bacteria; Table S5: Within-type PERMANOVA: effect of geographic region on the four indicator groups, tested separately within each fertilizer type; Figure S1: Nutrient content and physicochemical parameters in commercial organic fertilizers based on regions in China. (**a**) Organic matter; (**b**) total N; (**c**) total P; (**d**) available K; (**e**) available P; (**f**) C/N ratio; (**g**) pH; (**h**) EC. * *p* < 0.05, ** *p* < 0.01, *** *p* < 0.001; Figure S2: Concentration of antibiotics and heavy metals in organic fertilizers based on regions in China. (**a**) Total antibiotics; (**b**) tetracyclines; (**c**) macrolides; (**d**) fluoroquinolones; (**e**) Cu; (**f**) Zn. * *p* < 0.05, ** *p* < 0.01, *** *p* < 0.001; Figure S3: Count of total culturable bacteria and ARB in organic fertilizers based on regions in China. (**a**) Total culturable bacteria; (**b**) OTC-resistant bacteria; (**c**) SMX-resistant bacteria; (**d**) OTC- & SMX-resistant bacteria. * *p* < 0.05, ** *p* < 0.01, *** *p* < 0.001; Figure S4: Relative proportion of antibiotic-resistant bacteria to total culturable bacteria in organic fertilizers grouped by type and region across China. (**a**) OTC-resistant bacteria by type; (**b**) SMX-resistant bacteria by type; (**c**) dual (OTC & SMX)-resistant bacteria by type; (**d**) OTC-resistant bacteria by region; (**e**) SMX-resistant bacteria by region; (**f**) dual (OTC & SMX)-resistant bacteria by region. * *p* < 0.05, ** *p* < 0.01, *** *p* < 0.001; Figure S5: Spearman correlation between fertilizer indicators and bacterial abundance. (**a**) Absolute counts of total culturable bacteria (TCB) and antibiotic-resistant bacteria (ARB); (**b**) normalized antibiotic-resistant bacteria counts (ARB/TCB). Correlation strength was classified as weak (|ρ| < 0.3), moderate (0.3 ≤ |ρ| < 0.5), or strong (|ρ| ≥ 0.5). Significance: * *p* < 0.05, ** *p* < 0.01, *** *p* < 0.001 (nominal, uncorrected for multiple comparisons); BH-corrected significance is reported in the text; Figure S6: Absolute abundance of ARGs and the class 1 integron gene *intI1* in organic fertilizers, grouped by region across China. (**a**) *tetG*; (**b**) *tetL*; (**c**) *tetW*; (**d**) *tetX*; (**e**) *floR*; (**f**) *cmlA*; (**g**) *sul1*; (**h**) *sul2*; (**i**) *ermB*; (**j**) *lnuA*; (**k**) *aadA*; (**l**) *intI1*. Significance: * *p* < 0.05, ** *p* < 0.01, *** *p* < 0.001; Figure S7: Abundance of 16S rRNA gene in organic fertilizers, grouped by (**a**) Fertilizer type and (**b**) region across China. Significance: * *p* < 0.05, ** *p* < 0.01, *** *p* < 0.001; Figure S8: Normalized abundance of ARGs and the class 1 integron gene *intI1* in organic fertilizers, grouped by type. (**a**) *tetG*; (**b**) *tetL*; (**c**) *tetW*; (**d**) *tetX*; (**e**) *floR*; (**f**) *cmlA*; (**g**) *sul1*; (**h**) *sul2*; (**i**) *ermB*; (**j**) *lnuA*; (**k**) *aadA*; (**l**) *intI1*. Significance: * *p* < 0.05, ** *p* < 0.01, *** *p* < 0.001; Figure S9: Normalized abundance of ARGs and the class 1 integron gene *intI1* in organic fertilizers, grouped by region across China. (**a**) *tetG*; (**b**) *tetL*; (**c**) *tetW*; (**d**) *tetX*; (**e**) *floR*; (**f**) *cmlA*; (**g**) *sul1*; (**h**) *sul2*; (**i**) *ermB*; (**j**) *lnuA*; (**k**) *aadA*; (**l**) *intI1*. Significance: * *p* < 0.05, ** *p* < 0.01, *** *p* < 0.001; Figure S10: Redundancy analysis (RDA) of ARGs and environmental factors in organic fertilizers; Figure S11: Principal coordinate analysis (PCoA) of four fertilizer-borne risk indicator groups based on Euclidean distances of z-score standardized values. Each row denotes one indicator group: (**a**) and (**b**) nutrients and physicochemical properties (OM, TN, TP, available K, available P, pH, EC); (**c**) and (**d**) contaminants (Cu, Zn, and 14 veterinary antibiotics); (**e**) and (**f**) antibiotic-resistant bacteria (ARB); and (**g**) and (**h**) antibiotic resistance genes (ARGs). In the left column, samples are colored by fertilizer type; in the right column, by geographic region. Dashed ellipses denote 68% confidence regions. PERMANOVA (marginal Type III model, 999 permutations) indicated that fertilizer type consistently explained a larger fraction of compositional variation than geographic region across all four groups (R^2^_type_ = 0.20–0.38 vs. R^2^_region_ = 0.07–0.15). Supplementary_Data_S1.xlsx. References [54,55,56,57,58,59,60,61,62,63] are cited in the supplementary materials.

## Abbreviations

The following abbreviations are used in this manuscript:

ARGs	Antibiotic resistance genes
ARB	Antibiotic-resistant bacteria
OM	Organic matter
*mla* gene	Macrolide resistance gene, lincosamide resistance gene, and aminoglycoside resistance gene
