# Supplementary material for: Comparative Analysis of Commercial Organic Fertilizers Revealing Elevated Nutrient and Contaminant Loads in Pig Manure-Based Fertilizer
Source: Microorganisms. 2026 Oct 15;14(10):2364. doi: 10.3390/microorganisms14102364 (PMC13707988; doi:10.3390/microorganisms14102364)
Supplement: Supplementary file 1 [file microorganisms-14-02364-s001.zip › microorganisms-4558307-supplementary.pdf]

Comparative Analysis of Commercial Organic Fertilizers Revealing Elevated Nutrient and Contaminant Loads in Pig Manure-Based Fertilizer

Na Li [1,*], Jing Ye [1], Chaoyang Wang [1], Changxiong Zhu [2] and Hongna Li [1,*]

[1] Institute of Environment and Sustainable Development in Agriculture, Chinese Academy of Agricultural Sci-ences, Beijing 100081, China; yejing@caas.cn (J.Y.); 15116946231@163.com (C.W.)

[2] College of Environmental Sciences and Engineering, Hebei University of Science and Technology, Shijiazhuang 050018, China; zhuchangxiong@126.com

* Corresponding author

Email: lina06@caas.cn (N.L.); lihongna@caas.cn (H.L.)

Tel: 86-010-82109561

Address: Institute of Environment and Sustainable Development in Agriculture, Chinese Academy of Agricultural Sciences, Beijing, 100081, PR China.

Table S1 Detection frequency (%) of antibiotics in different types of organic fertilizers

Class	Antibiotics	Pig (n=10)	Chicken (n=12)	Cattle (n=9)	Sheep (n=12)	BOF (n=6)	Other (n=5)
Quinolones	Ofloxacin	100	100	100	100	100	100
	Norfloxacin	100	100	100	100	100	100
	Enrofloxacin	100	100	100	100	100	100
	Ciprofloxacin	100	100	100	100	100	100
	Danofloxacin	90	100	100	100	100	100
	Sarafloxacin	100	100	100	100	100	100
Tetracyclines	Tetracycline	10	100	77.8	80.3	14.3	0
	Oxytetracycline	10	100	11.1	0	0	0
	Chlortetracycline	50	0	11.1	91.7	0	0
	Doxycycline	100	0	33.3	58.3	0	20
Macrolides	Erythromycin	0	0	0	0	14.3	0
	Azithromycin	10	33.3	11.1	33.3	14.3	0
	Tylosin	0	8.3	11.1	8.3	14.3	0
	Tilmicosin	50	16.7	44.4	58.3	16.7	0
Sulfonamides	Sulfadoxine	0	0	0	0	0	0
	Sulfadiazine	0	0	0	0	0	0
	Sulfamethazine	0	0	0	0	0	0
	SMX	0	0	0	0	0	0
	Sulfamethazine	0	0	0	0	0	0

Class	Antibiotics	Pig<br>(n=10)	Chicken<br>(n=12)	Cattle<br>(n=9)	Sheep<br>(n=12)	BOF<br>(n=6)	Other<br>(n=5)
	Sulfamethoxypyrimidine	0	0	0	0	0	0
	Sulfamethoxazole-trimethoprim	0	0	0	0	0	0
	Sulfathiazole	0	0	0	0	0	0

Table S2 Gradient elution program

Time (min)	Phase A, %	Phase B, %
0.0	95	5
0.5	95	5
3.0	85	15
10.0	60	40
18.0	0	100
22.0	0	100
22.1	95	5
24.0	95	5
26.0	95	5

Table S3 Primer sets used in this study

Target	Sequence (5’-3’)	Length (bp)	Reference
16S rRNA	*F*-CGGTGAATACGTTC<br>*R*-AAGGAGGTGATCC	124	[20]
tetG	*F*-TTATCGCCGCCGCCCTTCT<br>*R*-TCATCCAGCCGTAACAGAAC	133	[54]
tetL	*F*-TCGTTAGCGTGCTGTCATTC<br>*R*-GTATCCCACCAATGTAGCCG	276	[55]
tetW	*F*-GAGAGCCTGCTATATGCCAGC<br>*R*-GGGCGTATCCACAATGTTAAC	168	[56]
tetX	*F*-AGCCTTACCAATGGGTGTAAA<br>*R*-TTCTTACCTTGGACATCCCG	280	[57]
floR	*F*-ATTGTCTTCACGGTGTCCGTTA<br>*R*-CCGCGATGTCGTCGAACT	190	[58]
cmlA	*F*-GCTGCTACTCCCCGTTAAGT<br>*R*-TGCCTGCCCATCATTAGTCC	117	[59]
sul1	*F*-CACCGGAAACATCGCTGCA<br>*R*-AAGTTCCGCCGCAAGGCT	158	[60]
sul2	*F*-CTCCGATGGAGGCCGGTAT<br>*R*-GGGAATGCCATCTGCCTTGA	190	[61]
ermB	*F*-CATGCGTCTGACATCTATCTGA<br>*R*-CTGTGGTATGGCGGGTAAGTT	190	[62]

Target	Sequence (5'-3')	Length (bp)	Reference
lnuA	*F*-GGTGGCTGGGGGGTAGATGTATTACTGG<br>*R*-GCTTCTTTTGAAATACATGGTATCGATC	323	[63]
aadA	*F*-CAGCGCAATGACATTCTTGC<br>*R*-GTCGGCAGCGACA(C/T)CCTTCG	293	[63]

Table S4 Spearman correlations between fertilizer indicators and bacteria

**Physicochemical**	**Resistant**	**ρ**	***p***	**adjusted *p***	**asterisks**
OM	OTC-resistant bacteria	0.133676	0.335213	0.744919	
Total N	OTC-resistant bacteria	0.040061	0.773642	0.927597	
Total P	OTC-resistant bacteria	0.177358	0.199486	0.664954	
Available K	OTC-resistant bacteria	0.134081	0.333741	0.744919	
Available P	OTC-resistant bacteria	0.360022	0.007495	0.282906	**
pH	OTC-resistant bacteria	0.064235	0.64447	0.927597	
EC	OTC-resistant bacteria	0.053783	0.699307	0.927597	
Cu	OTC-resistant bacteria	0.188184	0.172972	0.664954	
Zn	OTC-resistant bacteria	0.181704	0.188517	0.664954	
Antibiotics	OTC-resistant bacteria	0.016733	0.904407	0.927597	
OM	OTC- & SMX-resistant bacteria	-0.09975	0.472962	0.859932	
Total N	OTC- & SMX-resistant bacteria	-0.12529	0.366677	0.771951	
Total P	OTC- & SMX-resistant bacteria	0.148008	0.285484	0.720608	
Available K	OTC- & SMX-resistant bacteria	-0.01826	0.895726	0.927597	
Available P	OTC- & SMX-resistant bacteria	0.246279	0.072624	0.534235	

Physicochemical	Resistant	ρ	*p*	adjusted *p*	asterisks
pH	OTC- & SMX-resistant bacteria	-0.02531	0.85583	0.927597	
EC	OTC- & SMX-resistant bacteria	0.019859	0.88666	0.927597	
Cu	OTC- & SMX-resistant bacteria	0.208157	0.13093	0.654652	
Zn	OTC- & SMX-resistant bacteria	0.240252	0.080135	0.534235	
Antibiotics	OTC- & SMX-resistant bacteria	0.214408	0.119501	0.654652	
OM	SMX-resistant bacteria	-0.03465	0.80357	0.927597	
Total N	SMX-resistant bacteria	-0.0024	0.98625	0.98625	
Total P	SMX-resistant bacteria	0.029922	0.829937	0.927597	
Available K	SMX-resistant bacteria	0.01971	0.887505	0.927597	
Available P	SMX-resistant bacteria	0.161238	0.244113	0.716712	
pH	SMX-resistant bacteria	0.03713	0.78981	0.927597	
EC	SMX-resistant bacteria	0.158986	0.250849	0.716712	
Cu	SMX-resistant bacteria	0.064151	0.644902	0.927597	
Zn	SMX-resistant bacteria	0.048828	0.725871	0.927597	
Antibiotics	SMX-resistant bacteria	-0.09144	0.510782	0.886745	
OM	Total culturable bacteria	0.33212	0.014145	0.282906	*

**Physicochemical**	**Resistant**	**ρ**	***p***	**adjusted *p***	**asterisks**
Total N	Total culturable bacteria	0.245364	0.073726	0.534235	
Total P	Total culturable bacteria	0.272542	0.046171	0.534235	*
Available K	Total culturable bacteria	0.115021	0.407555	0.815109	
Available P	Total culturable bacteria	0.109514	0.430517	0.820032	
pH	Total culturable bacteria	0.187942	0.173535	0.664954	
EC	Total culturable bacteria	-0.03103	0.823749	0.927597	
Cu	Total culturable bacteria	0.147173	0.288243	0.720608	
Zn	Total culturable bacteria	0.065486	0.63802	0.927597	
Antibiotics	Total culturable bacteria	0.086908	0.532047	0.886745	

Note: This table provides the adjusted *p*-values (Benjamini–Hochberg FDR) for the Spearman correlations shown in the heatmap of Figure S5; the figure displays only the correlation coefficients (ρ) and significance asterisks, not the adjusted *p*-values. Fertilizer indicators comprise physicochemical properties, nutrients (N, P, K), heavy metals (Cu, Zn), and antibiotics. Significance: * $p < 0.05$, ** $p < 0.01$, *** $p < 0.001$ (based on adjusted p).

Table S5 Within-type PERMANOVA: effect of geographic region on the four indicator groups, tested separately within each fertilizer type

**Fertilizer type**	**Physicochemical**	**Contaminants**	**ARB**	**ARGs**
BOF	$R^2 = 0.22$, $p = 0.817$ R^2	$R^2 = 0.53$, $p = 0.100$	$R^2 = 0.20$, $p = 0.883$	$R^2 = 0.17$, $p = 0.950$
Pig	$R^2 = 0.20$, $p = 0.839$	$R^2 = 0.37$, $p = 0.315$	$R^2 = 0.42$, $p = 0.192$	$R^2 = 0.44$, $p = 0.236$
Chicken	$R^2 = 0.45$, $p = 0.127$	$R^2 = 0.26$, $p = 0.298$	$R^2 = 0.21$, $p = 0.588$	$R^2 = 0.27$, $p = 0.441$
Cattle	$R^2 = 0.38$, $p = 0.488$	$R^2 = 0.46$, $p = 0.273$	$R^2 = 0.13$, $p = 0.976$	$R^2 = 0.18$, $p = 0.863$
Sheep	$R^2 = 0.60$, $p = 0.003$*	$R^2 = 0.27$, $p = 0.480$	$R^2 = 0.30$, $p = 0.412$	$R^2 = 0.26$, $p = 0.529$
Other	$R^2 = 0.43$, $p = 0.700$	$R^2 = 0.33$, $p = 0.700$	$R^2 = 0.48$, $p = 0.700$	$R^2 = 0.25$, $p = 0.800$

Note: * $p < 0.05$. R^2 = proportion of variance explained by region within each fertilizer type (adonis2, Euclidean distance on z-scored variables, 999 permutations). ARB: antibiotic-resistant bacteria; ARGs: antibiotic resistance genes.

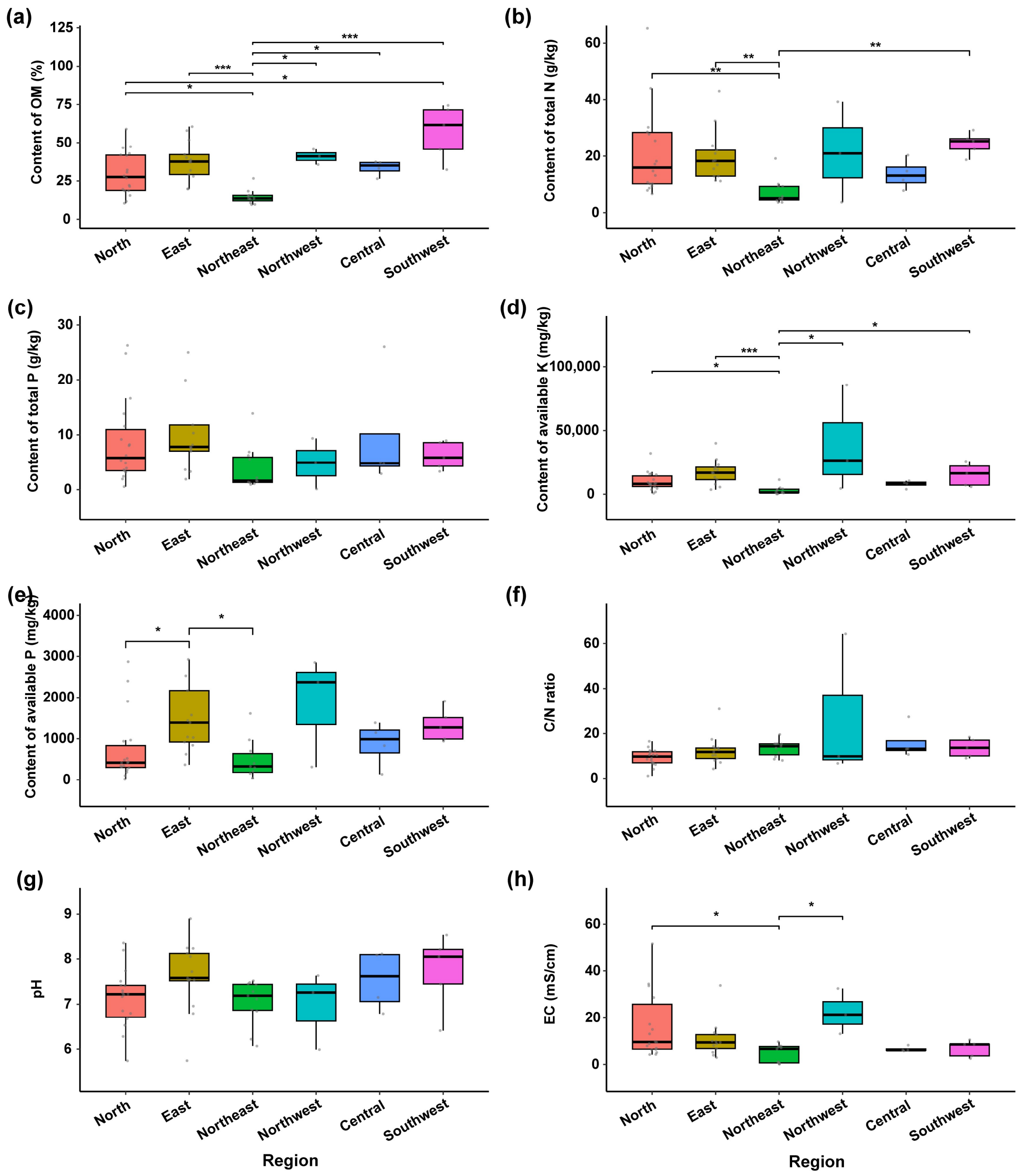

Figure S1. Nutrient content and physicochemical parameters in commercial organic fertilizers based on regions in China. (**a**) Organic matter; (**b**) total N; (**c**) total P; (**d**) available K; (**e**) available P; (**f**) C/N ratio; (**g**) pH; (**h**) EC. * $p < 0.05$, ** $p < 0.01$, *** $p < 0.001$.

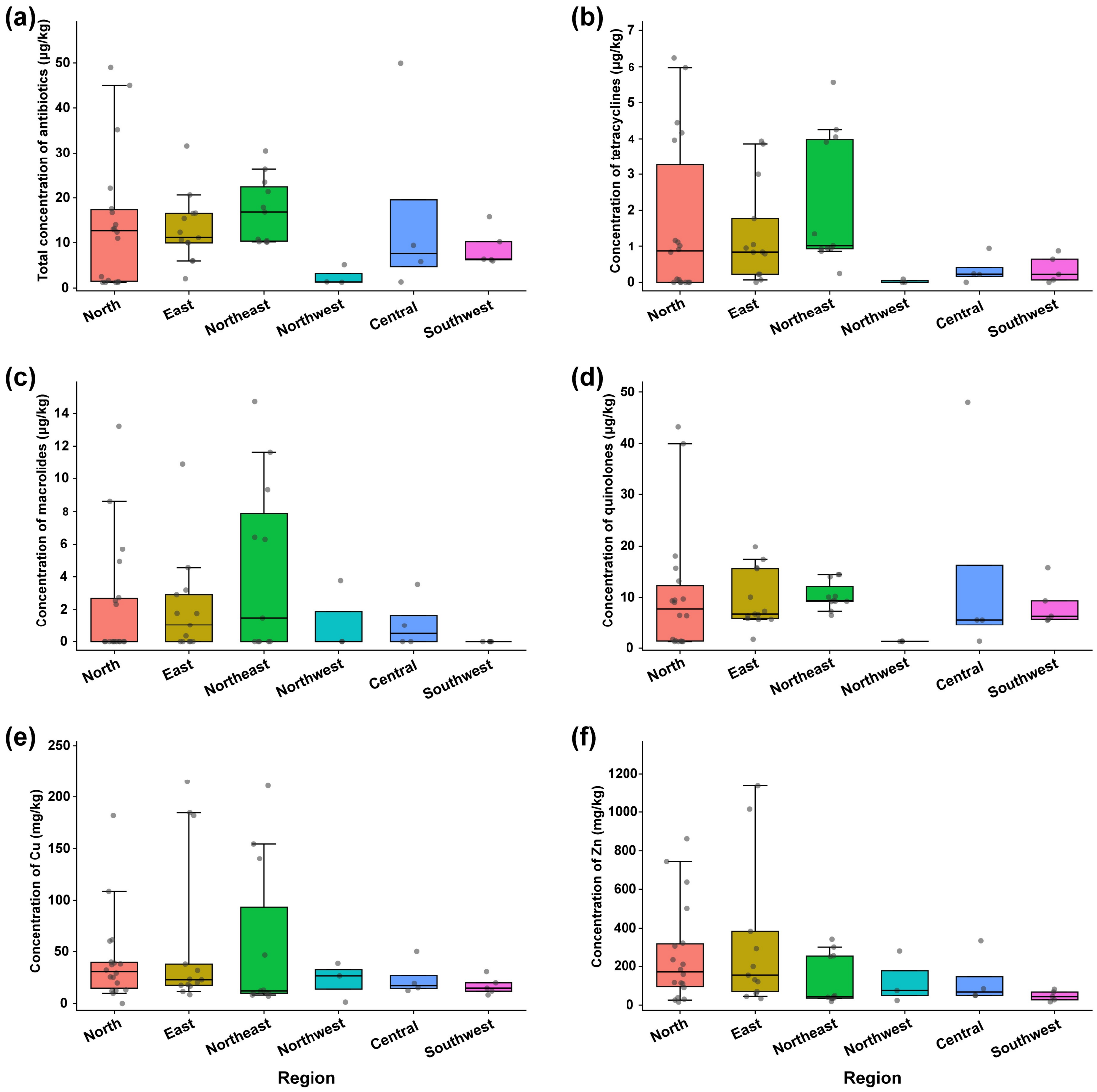

Figure S2. Concentration of antibiotics and heavy metals in organic fertilizers based on regions in China. (**a**) Total antibiotics; (**b**) tetracyclines; (**c**) macrolides; (**d**) fluoroquinolones; (**e**) Cu; (**f**) Zn. * $p < 0.05$, ** $p < 0.01$, *** $p < 0.001$.

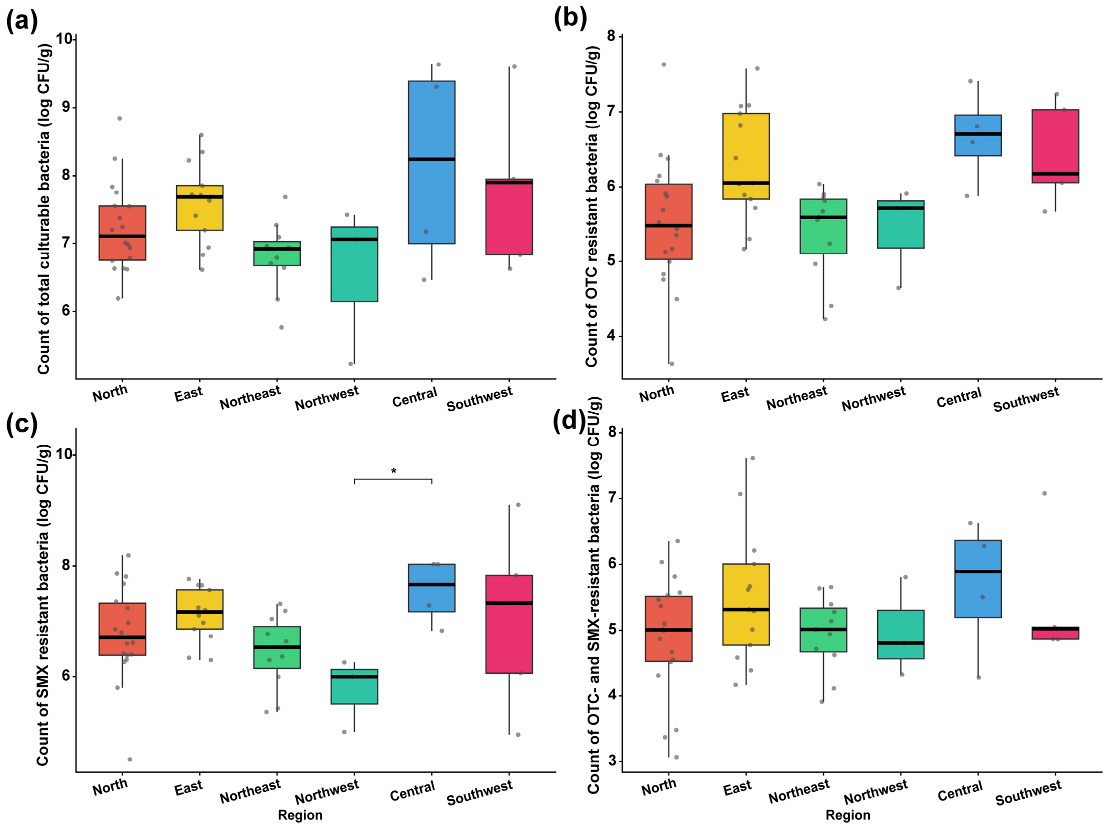

Figure S3. Count of total culturable bacteria and ARB in organic fertilizers based on regions in China. (**a**) Total culturable bacteria; (**b**) OTC-resistant bacteria; (**c**) SMX-resistant bacteria; (**d**) OTC- & SMX-resistant bacteria. * $p < 0.05$, ** $p < 0.01$, *** $p < 0.001$.

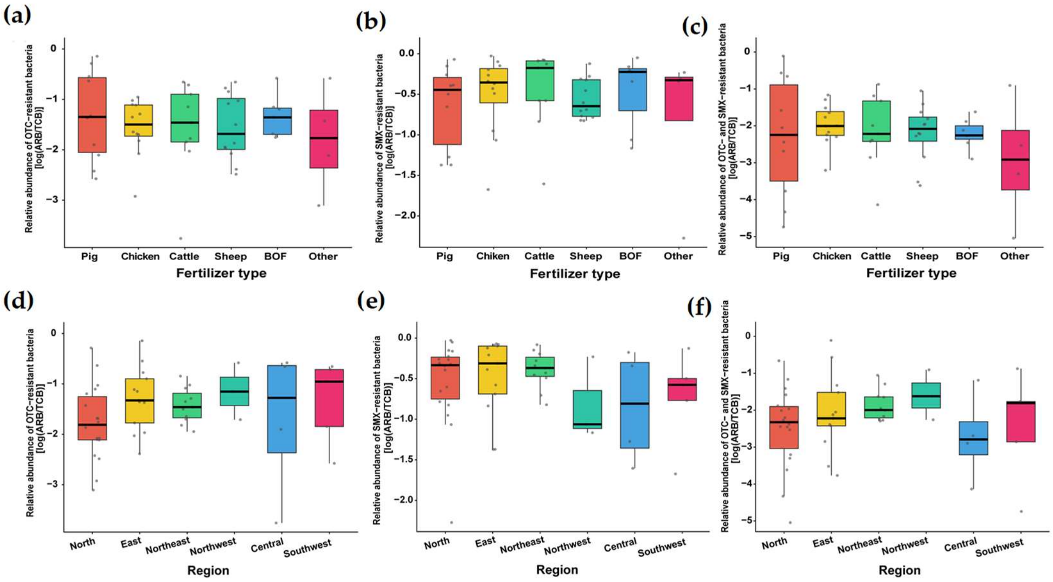

Figure S4. Relative proportion of antibiotic-resistant bacteria to total culturable bacteria in organic fertilizers grouped by type and region across China. (**a**) OTC-resistant bacteria by type; (**b**) SMX-resistant bacteria by type; (**c**) dual (OTC & SMX)-resistant bacteria by type; (**d**) OTC-resistant bacteria by region; (**e**) SMX-resistant bacteria by region; (**f**) dual (OTC & SMX)-resistant bacteria by region. * $p < 0.05$, ** $p < 0.01$, *** $p < 0.001$.

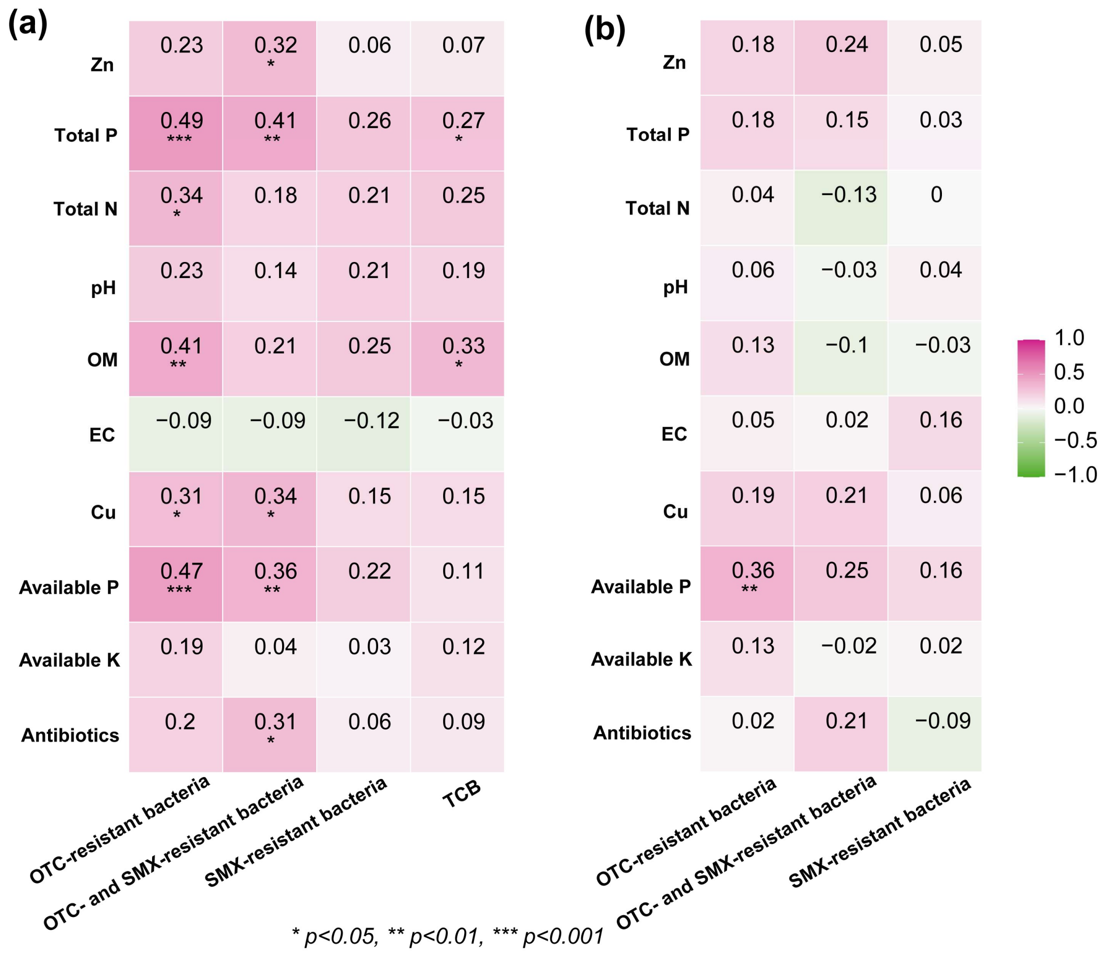

Figure S5. Spearman correlation between fertilizer indicators and bacterial abundance. (**a**) Absolute counts of total culturable bacteria (TCB) and antibiotic-resistant bacteria (ARB); (**b**) normalized antibiotic-resistant bacteria counts (ARB/TCB). Correlation strength was classified as weak ($|\rho| < 0.3$), moderate ($0.3 \leq |\rho| < 0.5$), or strong ($|\rho| \geq 0.5$). Significance: * $p < 0.05$, ** $p < 0.01$, *** $p < 0.001$ (nominal, uncorrected for multiple comparisons); BH-corrected significance is reported in the text.

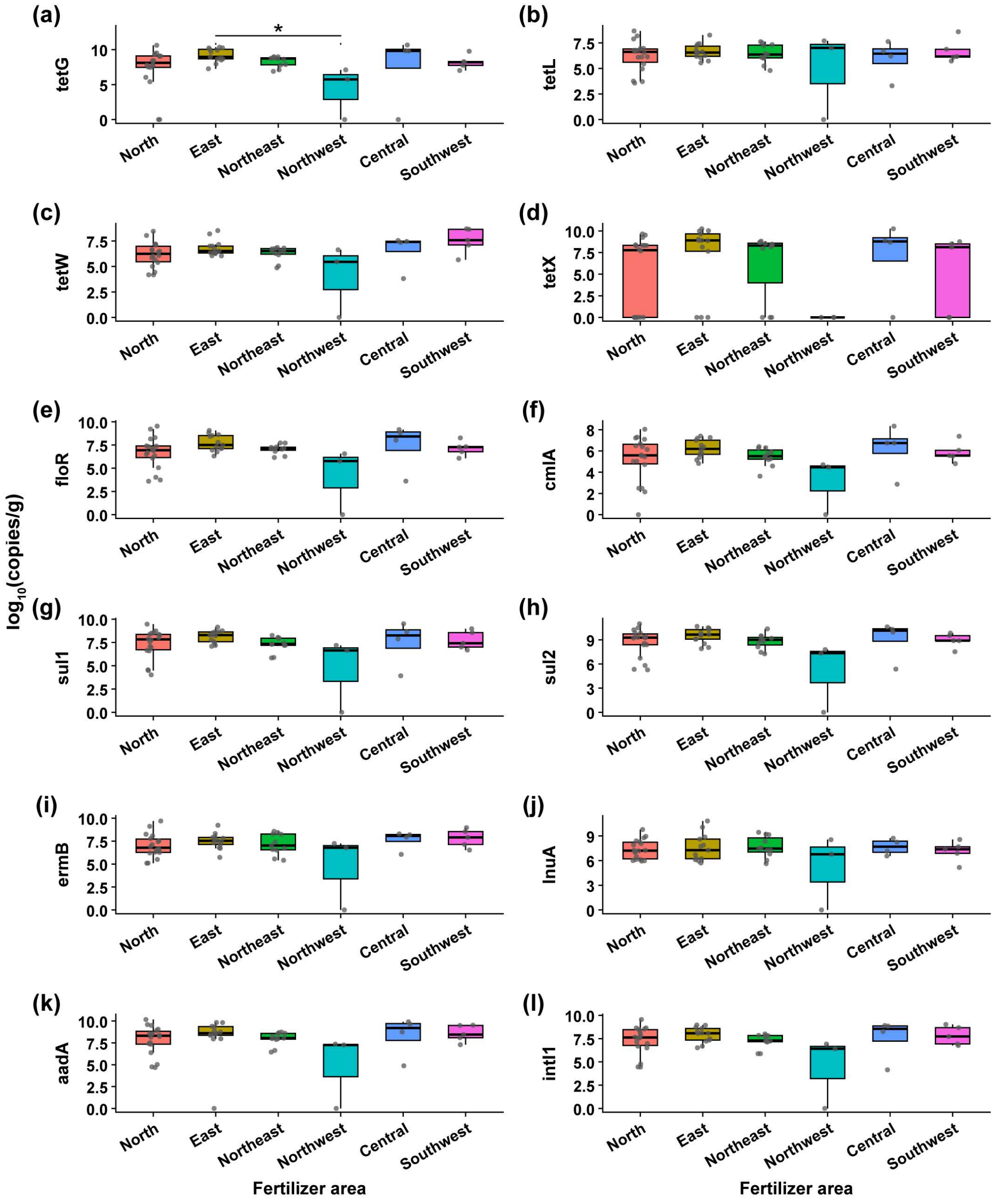

Figure S6. Absolute abundance of ARGs and the class 1 integron gene *intI1* in organic fertilizers, grouped by region across China. (**a**) *tetG*; (**b**) *tetL*; (**c**) *tetW*; (**d**) *tetX*; (**e**) *floR*; (**f**) *cmlA*; (**g**) *sul1*; (**h**) *sul2*; (**i**) *ermB*; (**j**) *lnuA*; (**k**) *aadA*; (**l**) *intI1*. Significance: * $p < 0.05$, ** $p < 0.01$, *** $p < 0.001$.

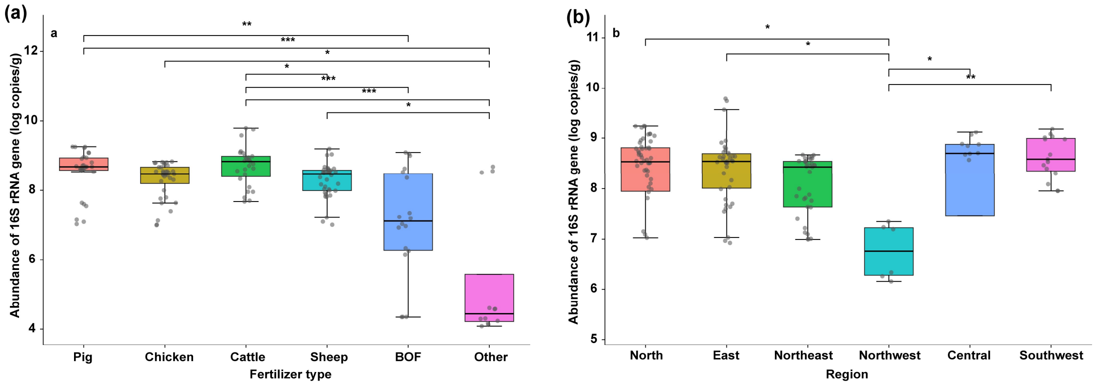

Figure S7. Abundance of 16S rRNA gene in organic fertilizers, grouped by (**a**) Fertilizer type and (**b**) region across China. Significance: * $p < 0.05$, ** $p < 0.01$, *** $p < 0.001$.

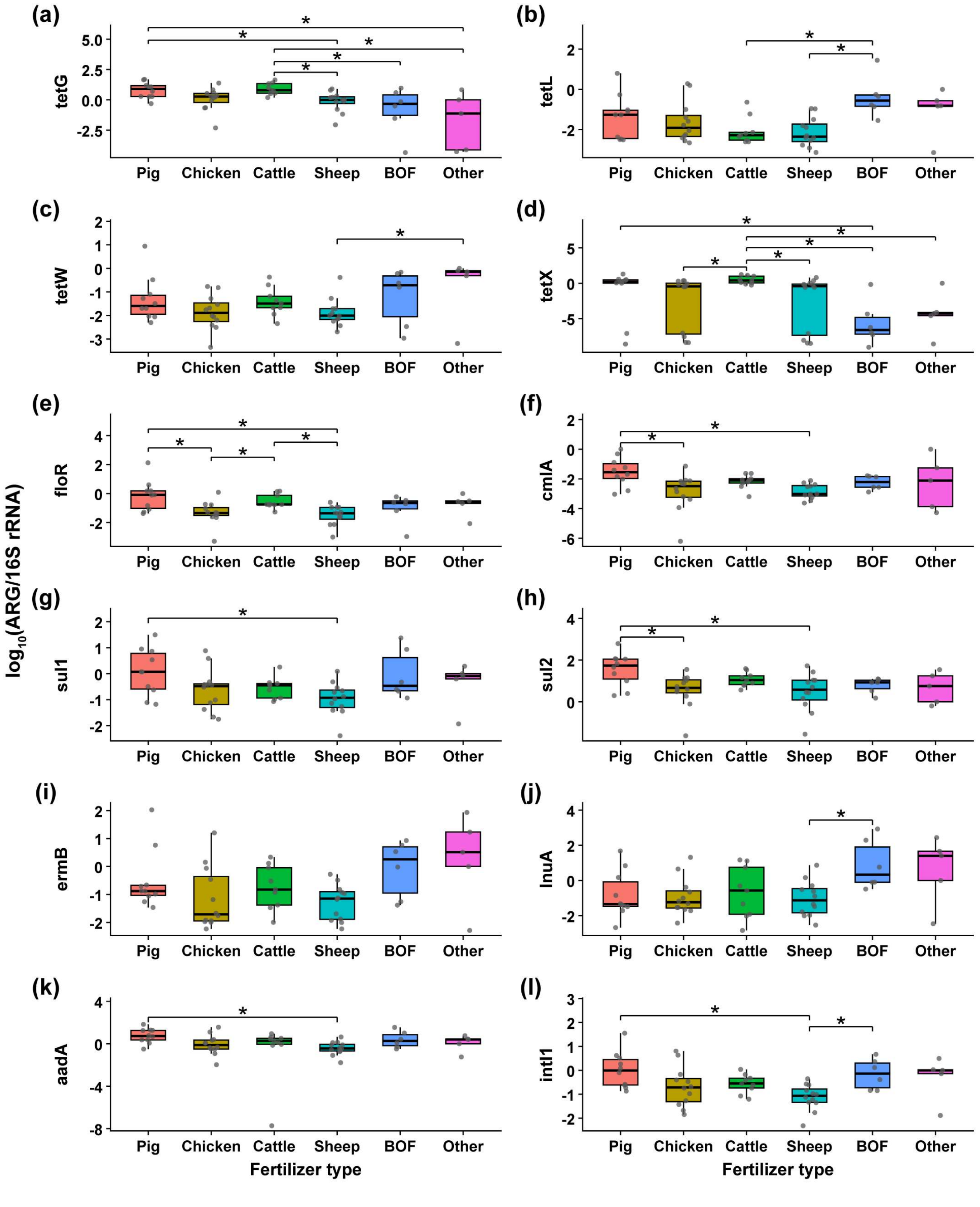

Figure S8. Normalized abundance of ARGs and the class 1 integron gene *intI1* in organic fertilizers, grouped by type. (**a**) *tetG*; (**b**) *tetL*; (**c**) *tetW*; (**d**) *tetX*; (**e**) *floR*; (**f**) *cmlA*; (**g**) *sul1*; (**h**) *sul2*; (**i**) *ermB*; (**j**) *lnuA*; (**k**) *aadA*; (**l**) *intI1*. Significance: * $p < 0.05$, ** $p < 0.01$, *** $p < 0.001$.

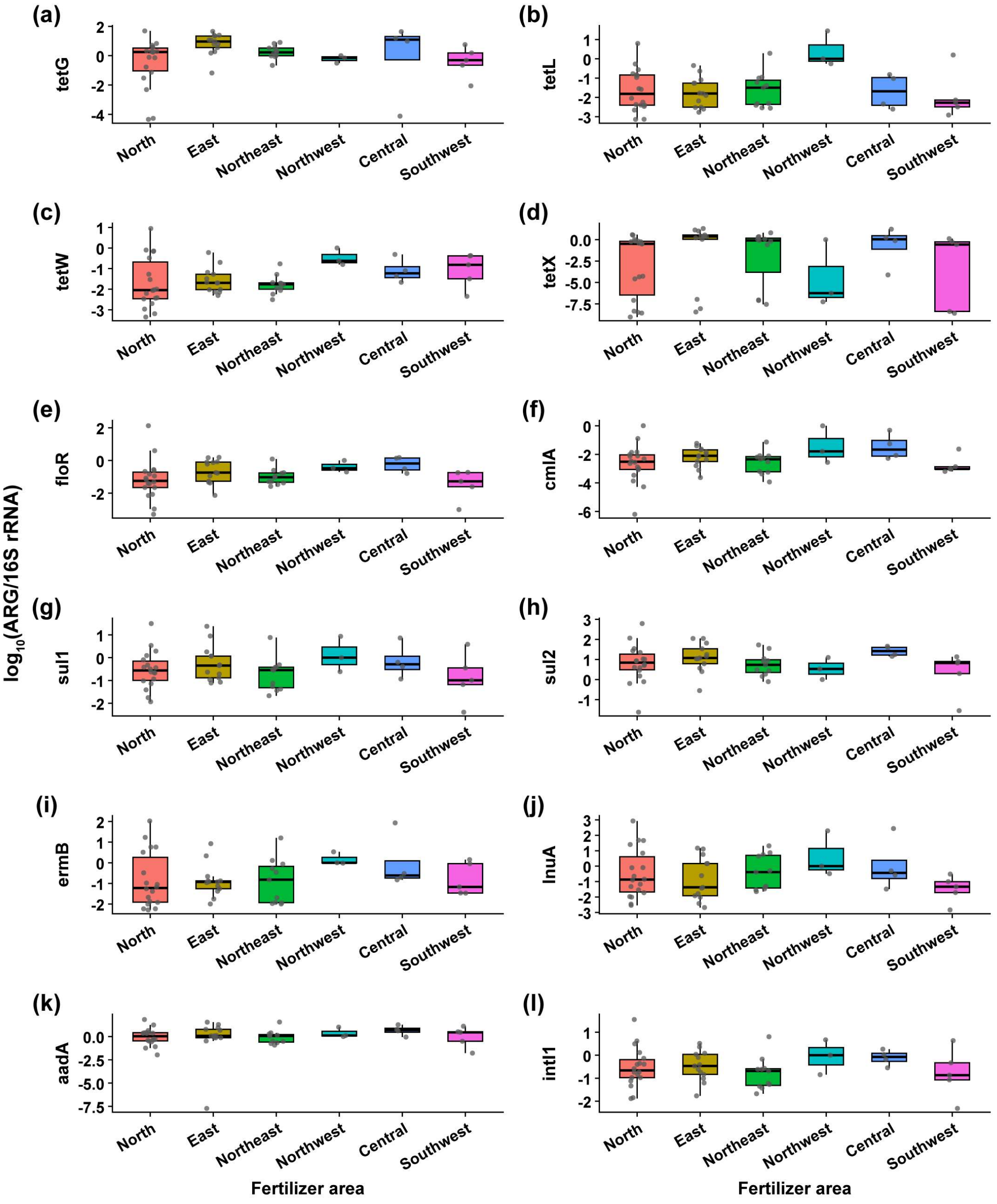

Figure S9. Normalized abundance of ARGs and the class 1 integron gene *intI1* in organic fertilizers, grouped by region across China. (**a**) *tetG*; (**b**) *tetL*; (**c**) *tetW*; (**d**) *tetX*; (**e**) *floR*; (**f**) *cmlA*; (**g**) *sul1*; (**h**) *sul2*; (**i**) *ermB*; (**j**) *lnuA*; (**k**) *aadA*; (**l**) *intI1*. Significance: * $p < 0.05$, ** $p < 0.01$, *** $p < 0.001$.

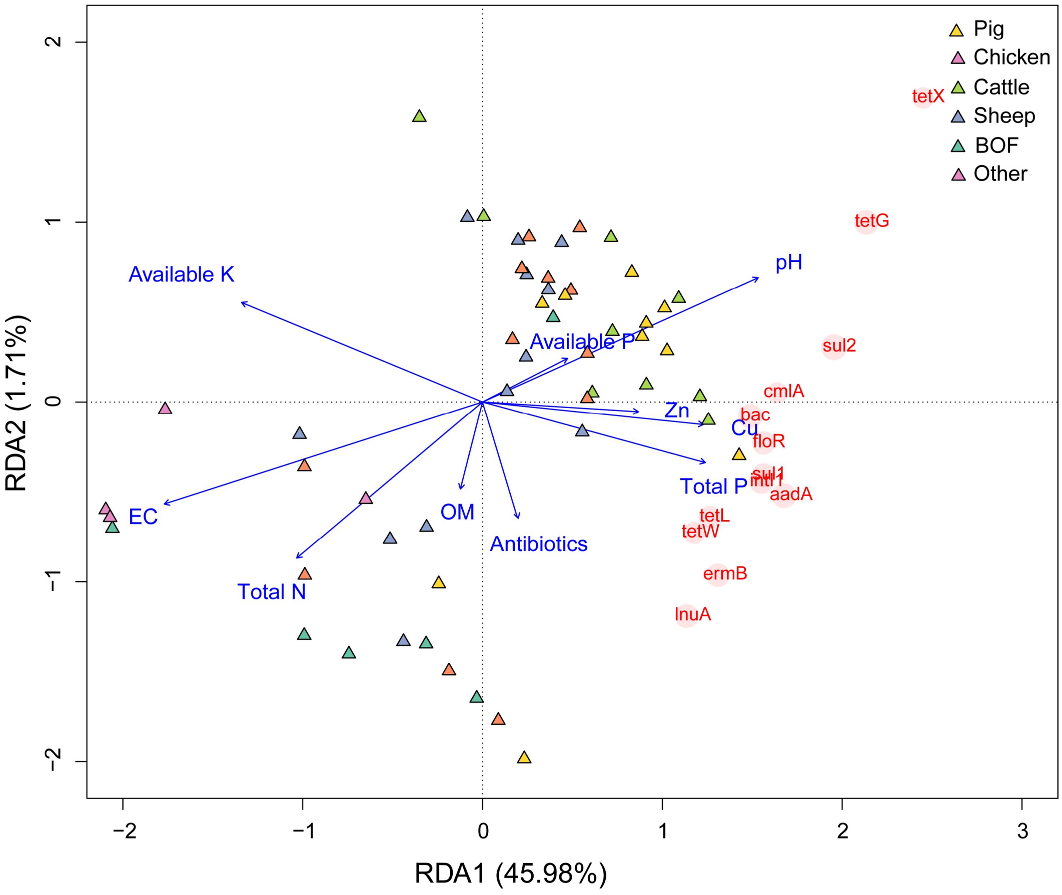

Figure S10. Redundancy analysis (RDA) of ARGs and environmental factors in organic fertilizers.

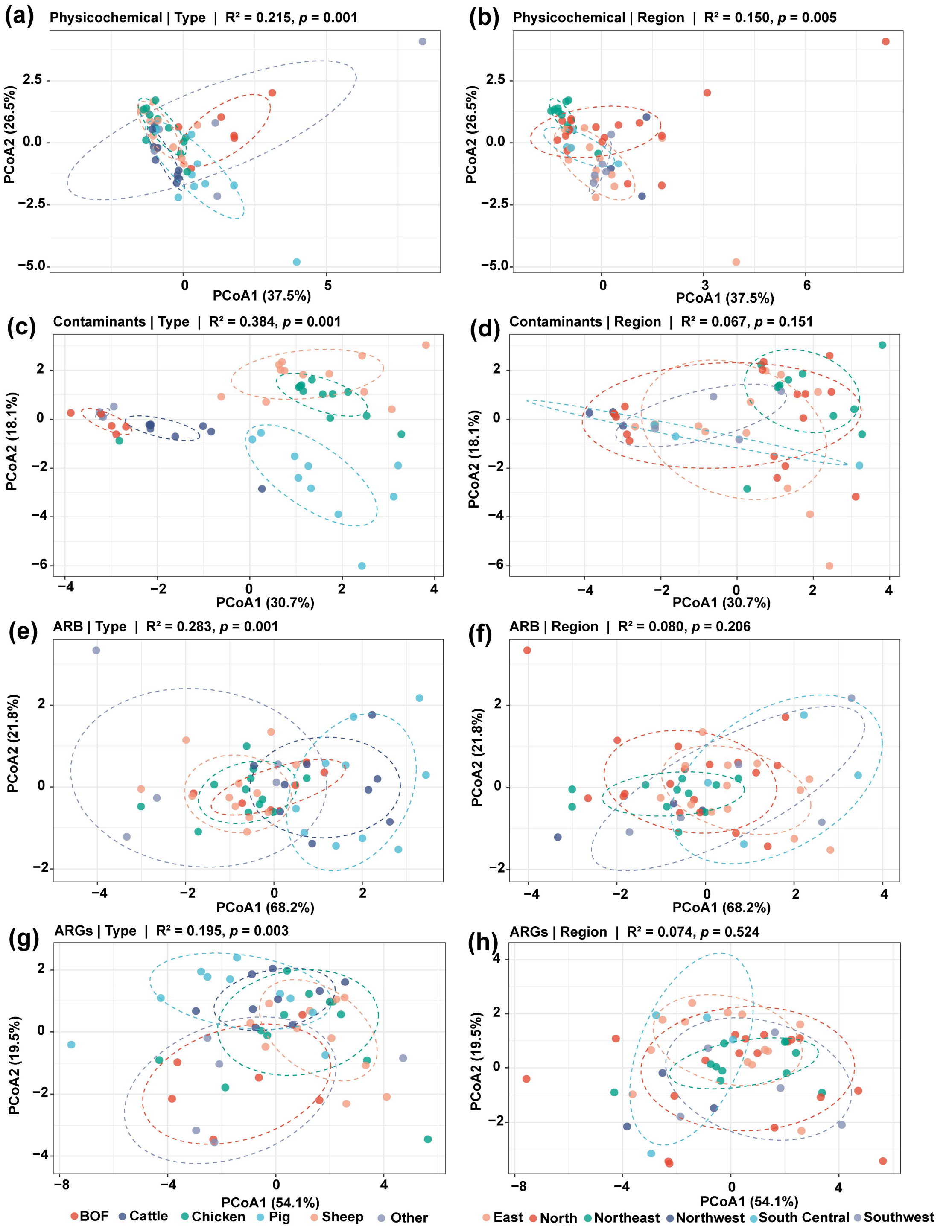

Figure S11. Principal coordinate analysis (PCoA) of four fertilizer-borne risk indicator groups based on Euclidean distances of z-score standardized values. Each row denotes one indicator group: (**a**) and (**b**) nutrients and physicochemical properties (OM, TN, TP, available K, available P, pH, EC); (**c**) and (**d**) contaminants (Cu, Zn, and 14 veterinary antibiotics); (**e**) and (**f**) antibiotic-resistant bacteria (ARB); and (**g**) and (**h**) antibiotic resistance genes (ARGs). In the left column, samples are

colored by fertilizer type; in the right column, by geographic region. Dashed ellipses denote 68% confidence regions. PERMANOVA (marginal Type III model, 999 permutations) indicated that fertilizer type consistently explained a larger fraction of compositional variation than geographic region across all four groups (R^2_{type} = 0.20–0.38 vs. R^2_{region} = 0.07–0.15).
